# How Personality and Communication Patterns Affect Online *ad-hoc* Teams Under Pressure

**DOI:** 10.3389/frai.2022.818491

**Published:** 2022-05-27

**Authors:** Federica Lucia Vinella, Chinasa Odo, Ioanna Lykourentzou, Judith Masthoff

**Affiliations:** ^1^Human Centred-Computing, Information and Computing Sciences, Utrecht University, Utrecht, Netherlands; ^2^The School of Natural and Computing Sciences, University of Aberdeen, Aberdeen, United Kingdom

**Keywords:** crowdsourcing, collaboration, social computing, personality, emergency response

## Abstract

Critical, time-bounded, and high-stress tasks, like incident response, have often been solved by teams that are cohesive, adaptable, and prepared. Although a fair share of the literature has explored the effect of personality on various other types of teams and tasks, little is known about how it contributes to teamwork when teams of strangers have to cooperate *ad-hoc*, fast, and efficiently. This study explores the dynamics between 120 crowd participants paired into 60 virtual dyads and their collaboration outcome during the execution of a high-pressure, time-bound task. Results show that the personality trait of Openness to experience may impact team performance with teams with higher minimum levels of Openness more likely to defuse the bomb on time. An analysis of communication patterns suggests that winners made more use of action and response statements. The team role was linked to the individual's preference of certain communication patterns and related to their perception of the collaboration quality. Highly agreeable individuals seemed to cope better with losing, and individuals in teams heterogeneous in Conscientiousness seemed to feel better about collaboration quality. Our results also suggest there may be some impact of gender on performance. As this study was exploratory in nature, follow-on studies are needed to confirm these results. We discuss how these findings can help the development of AI systems to aid the formation and support of crowdsourced remote emergency teams.

## 1. Introduction

Situations that require working together, fast, and efficiently under pressure are on the rise, especially in an increasingly fragile global ecosystem (Schneider, 2011; Kretzschmar et al., 2022). From handling widespread geopolitical conflicts (Friede, 2022) to mitigating environmental disasters (Gay-Antaki, 2021), several organizations are investing in crowdsourcing intervention to aid large-scale mobilization of resources including emergency shelters and disaster-event detection (Pettet et al., 2022; Stephens and Robertson, 2022; Zhang, 2022). Likewise, virtual teamwork enacted in high-urgency, high-stress tasks is on demand. Grassroots social engagement [i.e., Covid-19 pandemic hackathons (Colovic et al., 2022)], incident response squads (Palen et al., 2007), community response teams, and on-call software solution teams (Anderson, 2020) are all examples of ongoing large-scale collaborative efforts. Emergency teams are devolving into technology, and the internet, in particular, to enforce the timely resolution of complex problems within limited time frames, often under stress, and potentially with collaborators who have never worked together in the past. The benefits of working virtually and remotely are evident as shown by the thriving field of telemedicine with remote surgical teams aiding medical centers in coping with widespread pandemics (Etheridge et al., 2022). Nevertheless, little is known about the factors that can make or break such teams. In this study, we attempt to answer questions such as: *What are the personality characteristics that render high-stake online teams successful? Which skills, abilities, or socio-cultural elements are essential to consider while forming these teams? Are there any particular communication patterns that can serve as early signals of effective teamwork under stress?* Answering these questions is crucial to leverage available resources and intellect in critical situations. Although group research has since long investigated the effect of factors including personality, knowledge, skills, or socio-cultural facets on virtual teamwork (Kichuk and Wiesner, 1997; Krumm et al., 2016), few studies examine these characteristics on the specific problem of online collaboration strained by external—psychological or time-related—aspects.

Teams performing in rapid response environments do not perform similarly to “normal” teamwork settings. They are under pressure from the high-demand context under which they operate. The time-bounded nature of the task increases the chances of failure (Driskell et al., 2018). Characteristics of team performance in rapid-response, high-stress contexts are team members' ability to work in a team and personality traits (McManus et al., 2004; Subramaniam et al., 2010). However, to date, studies on high-stake teams focus either on emergency professional teams, crowd participation in emergency response, or the collaboration between these two groups without considering the aspect of team formation at the crowd level. Our study observes **remote, ubiquitous, online, and** ***ad-hoc*** **crowd teams** instead of traditional emergency response offline teams with specialized individuals (Chen et al., 2008). We deem the crowd, alongside teamwork emergency response, as the two most relevant aspects of this research, as we analyze and report properties contributing to successful outcomes under situations of stress and ambiguity. Furthermore, we examine the relationship between personality, socio-cultural elements, and communication patterns on the one hand, with team performance and satisfaction on the other, in the context of *ad-hoc online teams in rapid-response, high-pressure tasks*.

### 1.1. The Task: A Virtual Maze for Remote Crowdsourcing Emergency Teamwork

To study participant interactions in *ad-hoc* teams of strangers under pressure, we turn to crowdsourcing, and a custom-made task. Our task is inspired by the “Keep Talking Nobody Explodes” (Knuth, 2021) puzzle video game. Participants work in dyads, and their common mission is to defuse a bomb that is placed within a maze, by combining information that is unique to each one of them. One participant is assigned the role of the “Defuser”: they can “walk" inside the maze toward the bomb and defuse it, but they do not know where the maze walls are. The other participant is assigned the role of the “Lead Expert”: they have the map of the maze but they cannot walk in it. The Defuser and the Lead Expert must exchange information and actions, to defuse the bomb within a limited amount of time. The task has been designed to have the same critical characteristics as actual emergency response tasks, namely a high-demanding environment, enforced role division, performance pressure and stress.

#### 1.1.1. High-Demanding Environment

Instances of crisis constitute a large part of what emergency teams have to deal with and radically define their functional and structural properties. Demanding environments have critical requirements with tangible consequences for poor performance (e.g., accidents, errors, stress). By portraying the element of urgency in the form of a virtual bomb and increased time pressure (Bell et al., 2018) we focus on a single objective—reaching the bomb on time—and deliver the results of a study task that is critically cooperative and built for productive communication. In our setting, virtual crowd teams must deliver innovative solutions and deliver them quickly. The typical environmental constraints of high-demanding tasks (time, urgency, risks) command for independent, stable, role-defined teams sharing mutual trust, values, and focus. As we reduce and inter-mediate communication through digital means, we impose an even further reliance on mutual objectives, well-defined roles and obligations, effective communication, and commitment.

#### 1.1.2. Enforced Role Division

During cases of emergency, each team member has a distinct and specific role to play (Baldwin and Woods, 1994), which is typically a-priori and externally defined. Emergency and periods of crisis often create the need for established protocols of interaction respective to each part (Harrison and Connors, 1984). Although role division is typically fixed for these response units (e.g., medical, logistic, security, public relations, etc.), it must nonetheless be adaptable when facing unpredictable outcomes. By assigning strangers to pre-defined roles, we replicate a scenario where team roles are agreed upon yet flexible and interposed. Through well-defined roles and responsibilities, we evaluate the matching capabilities of crowd workers and investigate what are the constituents that fundamentally determine the execution of role-based virtual teamwork emergency response.

#### 1.1.3. Performance Pressure and Stress

Prior work has shown that users involved in games such as the crowdsourcing task exhibit various forms of stress (Sabo and Rajčáni, 2017) and heightened emotional states (Hart et al., 2018). These teams are more susceptible to allostatic load, i.e., the process of “wear and tear” experienced by team players facing stressful conditions (Davaslioglu et al., 2019). Regarding the definition of stress, there are two kinds of stressful conditions and stressors (Ma et al., 2021). One definition follows the general assumption that a stressor (the triggering factor) negatively affects the person by degrading performance; the other sees stress as a challenge that improves performance and individual gains (Zhang and Lu, 2009). In this research, we stripped the task from several elements of the original video game with the intent to transverse from multiple sources of hindering stressors [that increase environmental demands and exceed the available resources (Salas et al., 1996; Gardner, 2012)] to a unique challenge to inspire and motivate collaborators. Finally, virtual teams experience stress differently than offline ones as they tend to experience lessened social support (Su et al., 2012) which exacerbates predispositions to stress and anxiety (Tarafdar and Stich, 2021). For this reason, even though we adjusted the task to limit encumbrance, we still regard the individual and team response to a stressful task as the determining factor for whether personal characteristics and/or team compositions help handle the challenge successfully.

By engaging the players in this high-pressure challenge, we examine whether personality characteristics (Conscientiousness, Extraversion, Neuroticism, Agreeableness, and Openness) may make individuals more prone to cooperation under time pressure. We further evaluate which, if any, combination of personalities results in better than average team performance. Similarly, we examine whether additional factors such as the participants' socio-cultural background affect their actual ability to work together and their satisfaction with teamwork. Understanding the crowds perception of the collaboration (and not only performance) will help the development of AI agents to support their needs—and not only effectiveness—in times of crisis. Additionally, perceptions on the collaboration may provide insights into why certain teams are more effective than others, and what teams may be willing to work together again on the next task. Thanks to the heterogeneous data gathered during the experiment, we look at the dyadic communication to unravel indicators of a given team's potential to cope with a high-demanding task under time pressure.

A focus of this research is the impact of participants' personality on *ad-hoc* online teamwork, that is crowd-sourced, brief, and under pressure. We use the Big Five personality model (Goldberg, 1990), also known as the Five-Factor model, to model and comprehend the relationship between crowd workers' personality traits and their disposition for online teamwork in emergency contingencies. We selected the Big Five model as it is most commonly used for personality analysis [e.g., Highhouse et al., 2022; Ikizer et al., 2022; Mammadov, 2022] and for artificial intelligence systems that automatically adapt to personality [see (Smith et al., 2019) for a review of personality models used for personalization in persuasive technology, intelligent tutoring systems and recommender systems]. Additionally, many validated instruments exist to measure the Big Five traits, including the brief version of the Big Five Personality Inventory (Rammstedt and John, 2007) which we use in this paper. The Big Five model distinguishes between 5 traits^1^, each of which has multiple facets (see Table 1)

**Table 1:** Positive and negative facets of the BIG-5 personality traits (Neuman et al., 1999).

**Big five traits**	**Positive facets**	**Negative facets**
Extraversion	Social, talkative, assertive, active	Retiring, sober, reserved, cautious
Agreeableness	Good-natured, gentle,	irritable, suspicious,
	Cooperative, hopeful	uncooperative, inflexible
Conscientiousness	Self-disciplined, responsible,	lacking self-discipline, irresponsible,
	Organized, scrupulous	Disorganized, unscrupulous
Emotional stability	Calm, enthusiastic,	Anxious, depressed,
	Poised, secure	Emotional, insecure
Openness to experience	Imaginative, sensitive	down-to-earth, insensitive,
	Intellectual, curious	simple, narrow

### 1.2. Research Scope: Human Factors for AI Intervention in Crowdsourcing Emergency Response Teams

As work shifts to increasingly digitized spaces and connections between collaborators are made broader by mobile and ubiquitous computing, we consider evaluating ways to channel these resources to help remote, crowdsourced emergency teams. Identifying attributes and interactions used in emergency crises can help organizations and research improve upon methods for remote communication. Our knowledge of characteristics that contribute to virtual emergency response teamwork can inform artificial intelligent systems in assessing whether and how an individual can be part of a response unit with limited time and resources, and also, if multiple possible workers and tasks exist, who to use for the emergency response teams.

The rest of the paper is organized as follows. Section 2 presents and discusses related work, including an overview of traditional teams under pressure and crowdsourcing efforts in this domain, as well as the study hypotheses. Section 3 describes the study design, including participant sample and task design. Section 4 describes the metrics used to capture participants' demographic characteristics, Big Five personality traits, and ability (prior experience and self-perceived ability), as well as the metrics of teamwork, namely: collaboration quality and communication patterns. Section 5 presents the results. In Section 6 we discuss the implications of this work, its limitations, and possible extensions for the future. Finally, section 7 concludes the paper with key findings and closing remarks.

## 2. Related Work

### 2.1. Teams in Classical High-Demand, Time-Pressing Settings

#### 2.1.1. Operational Setting and Problem Scope

Significant research effort has been placed over the years on teams that need to perform in situations that require spontaneous, *ad-hoc* decisions and short-term planning, to resolve ambiguous or uncertain events, and where the consequences of failure are significant (Reuter et al., 2014). The scope of the problems that such teams are called to deal with is broad. It can include responding to natural disasters, like floods, hurricanes, and fires, but also managing crises (King, 2002), such as terrorism events (Longstaff and Yang, 2008), events occurring in long-duration spaceflights (Salas et al., 2015), nuclear plant control rooms (Stachowski et al., 2009), or situations taking place in a military context (Driskell et al., 2014). It can also include more benign everyday workplace settings, such as on-call software teams dealing with organizational incidents, like security or service failure events (for example the recent Google outage (Bergen, 2020), journalist teams for the immediate coverage of unexpected events (Archibold, 2003), but also short-term project teams (Galbraith and Lawler, 1993) and task forces (Hackman, 1990). Their size can vary, from dyads and triads (Foushee, 1984), to dozens (Helmreich, 1967), to twenty or more (Stuster, 2011).

#### 2.1.2. Differences From Normal Teams

What separates these teams from teams in “normal” settings, is the extreme, atypical environment within which they operate, which overall entrails time pressure, high levels of risk, increased consequences for poor performance (Driskell et al., 2018), no previous work experience with one another, and the need to perform their task almost immediately on team formation (Mckinney et al., 2005; Mendonça, 2007). Harrison and Connors (1984) use the term exotic environment to describe a work setting that is marked by hostile environmental demands, restricted working conditions, isolation from those outside the setting, and confinement and enforced interactions for those inside it. Using the related term extreme environment, Bell et al. (2018) add that these settings are also characterized by limited time to finish the task. Performance pressure and severe consequences for ineffective performance are also characteristic of these settings, and this pressure can act as a double-edged sword that can lead the team to outstanding performance, or cripple it Gardner (2012). The tasks that teams in these settings must solve are usually characterized by ambiguity and urgency (Yu et al., 2008; Stachowski et al., 2009).

#### 2.1.3. Factors Affecting the Success of Emergency Teams

Which factors determine team success in this high-demand, high-stress environment? *Skill* and expertise are the primary factors. Teams traditionally trained as emergency response units rely on the specialized expertise of the stages of the incident response and carry insider knowledge of the organizational policies, their obligations, the communication channels, and the tools supplied by the hiring organization. Thereof, the effectiveness of traditionally formed emergency response teams relies to a great extent on the level of preparedness and competence of the hiring body (or authority) that trained and assembled them, with multiple historical incidents providing evidence for the need for precise training programs and hiring criteria (Alexander, 2003). Examining command and control teams, Ellis et al. (2005) find that team members with higher training demonstrated greater proficiency in planning and task coordination activities, as well as in collaborative problem-solving, and communication. The study also found that it is the knowledge competencies of the team member with the most critical position that benefited the team the most.

The second factor of interest is the allocation of *roles and authority*. A prominent characteristic of typical high-stake teams, such as STAts (swift-starting action teams), is that they comprise experts (Mckinney et al., 2005) with specific roles and responsibilities. Multiple studies confirm the value of stable role structure in the division of labor and in enhancing the predictability of team interactions, allowing each team member to know what to expect from their teammates in critical situations (Hackman and Morris, 1975; Stachowski et al., 2009). The reason is that misunderstandings or disagreements about authority and role accountability (especially non-desirable roles like clean-up) may lead to team conflict, especially in the presence of unprecedented emergency response tasks (Quarantelli, 1988; Weick, 1993). The meta-analysis of De Wit et al. (2012) further confirms the negative relationships between process and role conflict, and team results such as cohesion, commitment, and performance. On the other hand, flexibility, the ability to improvise, and entrusting functional requirements to determine roles, rather than relying on titles may also be of benefit (Briggs, 2005; Mendonça, 2007). A highly defined role structure with clear roles seems to benefit more tasks that are structured. On the contrary, a flatter structure may be better for ambiguous tasks for which no apparent solution can be easily found (Worchel and Shackelford, 1991) (such as the task of responding to the 2001 World Trade Center attack Mendonça, 2007).

*Personality* is another prominent factor affecting the success of high-stakes teams, in line with the broader personnel selection literature which indicates that if relevant personality factors are identified for a specific job, future performance can be predicted (Borman et al., 1980). Using the occupational personality questionnaire to study the emergency command ability of offshore installation managers, Flin and Slaven (1996) finds significant correlations between command abilities in critical situations and certain personality elements. From their results, it appears that the highest-rated performance came from those who (a) like to take charge and supervise others (high score on controlling), (b) consider themselves to be fun-loving, sociable, and humorous (high score on outgoing), (c) are less interested in analyzing human behavior (low score on behavioral), (d) are more interested in practical than abstract problem solving (low score on conceptual), and (e) prefer to make decisions quickly rather than take time to weigh up all the evidence (high score on decisive).

Flin and Slaven (1996) contribution, however modest in size, is only pertinent to emergency command responsibilities and applicable only within a specific type of organization (offshore installation managers). Other researchers have focused on the possible existence of a “rescue personality,” in multiple additional domains where emergency services and occupational stress are pivotal. Kennedy et al.'s (2014) research on how personality influences the workforce decisions of emergency nurses reveals that certain traits matter more than others. High Extraversion, Openness to experience, and Agreeableness were especially common amongst emergency nurses. Extraversion was also present among emergency department senior medical staff (Boyd and Brown, 2005) as part of the controversial ENTJ (Extrovert, Intuitive, Thinking, Judging) personality type^2^ (Myers, 1962).

Partially supporting these findings is the work of Wagner et al. (2009) on the personality traits of paid professional firefighters. Although high Conscientiousness was not a determinant factor in this vocational role, Extraversion had significance. Certain personality traits seem to cluster under particular types of emergency professions; the differentiation between correlation and causality between these two variables is not always easy to untangle. Feelings of anxiety and insecurity, as well as heightened levels of Neuroticism and Openness, were seen to be most likely the results, and not the cause, of the repetitive exposure to experiences of loss and distress (Pajonk et al., 2011). By broadening the sample to the general public (virtual crowd), we aim at decoupling the effects that a specialized profession could have on one's propensity to emergency response.

Finally, certain *interaction patterns* are useful predictors of whether an *ad-hoc* team that has been brought together for immediate task performance will succeed or not, in classical emergency response teams. Although swift-start teams have little time to build their group processes before starting to work on the task, it is also known that team routines get established early in the team's lifecycle. The same initial interactions have an effect on subsequent communication and norms (Gersick and Hackman, 1990). The study of Zijlstra et al. (2012) reveals that there are certain early patterns of communication that distinguish effective from less effective teams. Specifically, they find that effective teams engage in communication that is more stable in duration and complexity, more balanced, and less monopolized by a single participant compared to inefficient teams that exhibit frequent mono-actor patterns, consisting of a single team member posing and answering their questions and commenting on their observations. They also found that efficient teams exhibit more reciprocity and trust, with the team members engaged and in the same direction of action toward the task goal. The presence of trust as a crucial factor is also highlighted (Wildman et al., 2012). The study of Waller et al. (2004) reveals that efficient teams in non-routine situations focused their actions on information collection and task prioritization. Finally, Kanki et al. (1991, 1989) complement the above by showing that the communication of effective swift-start two-person crews focuses on immediate task execution, expressed as low-complexity, straightforward action statements, and is less focused on other non-standard communication.

Although classical rapid-action teams are widely studied, these literature findings do not necessarily translate to online crowd rapid-action teams. Traditional emergency teams comprise highly trained professionals with a shared understanding of the crisis domain, and often a shared loyalty to an organization. In contrast, crowd teams mainly consist of non-experts, and they are more volatile and heterogeneous regarding the motivators that draw their members to the particular task. Considering the multiplication and globalization of the events that require swift action, it is likely that in the future, we will need to turn more and more to crowd workers and volunteers to form *ad-hoc* online teams that can deal with high-stake situations under pressure. In this light, the extensive study of classical rapid-action teams can provide us with the first grounded indications of specific parameters to look at to identify predictors of successful team formation in online crowd action teams. Given that in a crowd setting, the allocation of roles is likely to take place based on arrival and availability, in this work, we focus on the parameters of personality and communication patterns as predictors of forming a successful crowd team to tackle unforeseen situations under time pressure.

#### 2.1.4. Onsite and Offsite Emergency Response Teams

The history of emergency response teams—and more broadly of emergency preparedness—is essentially as old as societal and humanitarian threats. For as long as emergencies have affected human lives, societies have found collective ways to organize efforts to mitigate, prepare, respond, and recover from the aftermaths of crises. Emergency preparedness programs have evolved along with societal changes and technological advancements. Notable historical events such as the first world war brought national societies to unify and strengthen their approaches to natural, intentional, and accidental disasters (Herstein et al., 2021). The International Federation of Red Cross and Red Crescent Societies is one of the most prominent products of global pursuits unifying volunteer networks, community-based expertise, and independent advisers into standardized practices (London, 1998). As emergency response evolves, emergency response teams reshape ways to communicate and function in an era of accelerated technological progress.

Formerly, emergency teams operated face-to-face and on-site in response to environmental disasters (Brennan and Flint, 2007), war conflicts (Abdul-Razik et al., 2021), and epidemics (Leach et al., 2022). With the broadening digitization of services, society is increasingly reliant on technology for its functioning. The so-called information era entails the vast market of the internet of things, software, and the worldwide web to enable widespread financial and data transactions (Stehr, 2001). Technological dependency is making us faster and smarter and, at the same time, more vulnerable to novel threats (e.g., malware attacks, identity theft, financial fraud, security breaches, etc.). Emergency response teams not only must face novel and extensive digital threats but must also learn to leverage the resourcefulness of recent technology [ubiquitous computing (Smirnov et al., 2011), robotics (Kawatsuma et al., 2012), simulations (Kincaid et al., 2003), smart sensors (Abu-Elkheir et al., 2016), and social media networks Potts, 2013] to strengthen their outreach and preparedness.

Overall, the vast majority of emergency response teams operate in a hybrid fashion combining onsite support with online offsite communication. Some others divide efforts between online and face-to-face tasks depending on the phase of the response (i.e., mitigation, preparedness, response, and recovery Brennan and Flint, 2007). Relevant to our research is the pertinence of virtual communication channels in the large-scale crowdsourced emergency response domain that is typically remote, collaborative, and online. To define our target group, we firstly identify general characteristics that, in the classical sense, differentiate between onsite and offsite emergency response teams. Although the two domains share very similar objectives and attributes such as organizational culture, expertise, team structure, communication, and teamwork (Leach and Mayo, 2013), since their capabilities and duties differ, some of these attributes are more imperative than others. In the following subsections, we introduce two representative attributes critical for each teamwork domain.

##### 2.1.4.1. Onsite Emergency Response Teams

Two prominent attributes of onsite teams are **experience** and **coordination**. Teams working onsite are usually part of rescue operations (Chen and Miller-Hooks, 2012) and disaster relief (Bjerge et al., 2016) that require the participation and coordination of experts. These include fire and rescue services and police forces, commercial entities, volunteer organizations such as the Red Cross, media organizations, and the public (Yang et al., 2009). The need for distinct expertise requires teams to develop and apply specialized knowledge. Onsite emergency response experts can hold intelligence on chemical properties, procedures for reporting emergencies, fire and protective equipment, decontamination, and evacuation gained through training, experience, and/or formal education.

Without qualified knowledge and standardized procedures, onsite emergency response teams would fall short of promptly and accurately addressing ongoing crises. Equally important is coordination among experts as onsite emergency must successfully distribute superintendence and responsibilities between diverse professionals for effective prevention, preparedness, and response to emergencies. In their work on coordination in emergency response management, Chen et al. (2008) developed a life-cycle approach with three distinct sets of activities on the timeline continuum (pre-incident phase, during incident phase, and recovery phase). The cycle closes after de-briefing and when actionable items are learned from the intervention and incorporated into the plan to affect future preparedness (Chen et al., 2008). The same authors identified several elements of coordination such as activities, coordination objects, and constraints that differ between phases and between cultural, political, regulatory, and infrastructural properties of emergency response.

##### 2.1.4.2. Offsite Emergency Response Teams

Two distinguishing attributes of offsite remote emergency response teams are **communication** and **sensemaking**. While onsite teams converge in rescue operations and disaster relief, remote offsite emergency response teams outreach and distribute resources. Known crises overseen by offsite emergency response teams are air-traffic control (Hughes et al., 1992), subway crisis management (Heath and Luff, 1992), and emergency response call centers (Normark, 2002; Pettersson et al., 2004). Although clear roles are important in these teams, clear communication is of the essence. Depending on the kind of interaction (e.g., serendipitous, inbound, and outbound Landgren and Nulden, 2007), and the referent (e.g., non-experts' communication, situation update, situational awareness, services access assistance Velev and Zlateva, 2012), clear communication and interaction protocols fundamentally determine the interaction mediated by computer systems for offsite rescue teams.

Through clear communication, offsite emergency response teams can harvest sensemaking. This is the collection of actions that make the situation understandable and that prevent an escalation of the emergency (Landgren and Nulden, 2007). Sensemaking has properties such as identity construction, retrospection, enactment, social reactions, dynamism, environmental cues, and plausibility (Muhren et al., 2010). The importance of sensemaking in a remote emergency context is ever so apparent due to the practical constraints that teams experience as they communicate remotely. According to Weick (1993), most shortcomings from failed emergency responses are due to a deficiency in sensemaking (or contextual rationality). Weick (1993)'s work uncovers four potential sources of resilience that make *ad-hoc* groups less vulnerable to disruption of sensemaking. These sources are (i) improvisation, (ii) virtual role systems, (iii) the attitude of wisdom, and (iv) norms of respectful interaction. Weick (1993) analyses the dynamics of role structure and sense-making occurring in the historical Mann Gluch disaster. The incident served as an example of what needs to be re-examined about temporary systems, structuration, non-disclosed intimacy, inter-group dynamics, and team building (Weick, 1993), especially important for offsite emergency response operations.

The design of computer-mediated emergency response also needs to be informed by an understanding of the cognitive processes involved in responding to unanticipated contingencies (Mendonça, 2007). These cognitive factors, defined by Mendonça (2007), are directly linked to the specificity of emergence management and its characteristics of rarity, time pressure, uncertainty, high and broad consequences, complexity, and multiple decision making. Besides, computer-mediated emergency response teams are much more predisposed to incorporate the output of citizen convergence (Schmidt et al., 2018) into their work than traditional onsite rescue teams. However, as developments in online informational convergence change the remote domain of rescue operations, citizens and crowds are bringing novel paradigms. These include unfamiliar team members, ill-defined tasks, fleeting membership, multiple and conflicting goals, and geographically distributed collaboration (Majchrzak and More, 2011). In the following section, we explore the topic of crowdsourcing for emergency response.

### 2.2. Crowdsourcing for Emergency Response

#### 2.2.1. Emergency Response Through Individual Crowd Contributions

Crowds are increasingly involved in response to emergencies. The characteristic of emergency response crowdsourcing is the short-lived engagement in the task. Crowds' contributions consist of primarily individual, one-time, and remote interactions. This “long-tail” of contributions is a well-observed phenomenon in most content-oriented online communities (Shirky, 2008). The role of these one-time crowd users is important when it acts as a fast and ubiquitous response to urgent, environmental and social crises (hurricanes, terrorist attacks, widespread fires, large oil spills, etc.) (Heinzelman and Waters, 2010; Yuan and Liu, 2018; Chau, 2020), protest movements (Elsafoury, 2020), but also activism (Farkas and Neumayer, 2017; Lee, 2020) and civic participation (Hemphill and Roback, 2014; Mitchell and Lim, 2018). In critical scenarios of this kind, the crowd is intended as a manifold social tool by servicing as a reporter, social computer, sensor, and executor of both micro and macro-tasks.

Several theoretical studies propose system models and features designed to facilitate the positioning of the crowd as the leading resource for emergency management. In the domain of communication technologies for health care Hossain et al. (2017) suggest benefiting from the users' social contacts to trigger a faster response, or to make the most of crowdsourcing attributes—such as collaboration and tournaments—to attract the right crowd for the job. From a complex systems perspective, Song et al. (2020) propose harnessing the self-organizing operation mechanisms of crowdsourcing for efficient disaster governance. In the context of natural disaster management, Ernst et al. (2017) propose hybrid systems that rely on the remote coordination of volunteers to collect location-dependent information, which in turn can support emergency managers making quick but solid decisions. Elsafoury (2020) propose another hybrid feature, this time combining machine learning with crowdsourcing to rapidly detect protest repression incidents through social media.

Specific crowdsourcing tools and platforms address emergencies. Poblet et al.'s (2013) review indicates that these platforms belong to two main categories, namely: (i) data-oriented, and (ii) communication-oriented. The first category concerns tools developed for the intensive aggregation, mining, and processing of data gathered through the crowd. The second category aims at supporting communication between crowd users and disaster management systems by allowing seamless interaction between them. The platform “Ushahidi” (Okolloh, 2009) is one example of a crowd application designed to decentralize the support of volunteers for the report of violence in Kenya, by collecting sensitive reports, organizing rapid response actions across multiple agencies, documenting ongoing changes, generating automatic alerts from under updates and visualizing data streams in real-time.

In another example, several digital volunteer organizations (Standby Task Force, Humanity Road, and Open Crisis) have integrated social media monitoring in their systems when cooperating with other humanitarian bodies in disaster relief operations (Poblet et al., 2013) Poblet et al.'s (2013) review of crowdsourcing tools for disaster management offers an extensive list of crowdsourcing tools, including online platforms and mobile applications across the globe. Aside from those tools that support response and recovery-based only efforts, others, such as ArcGIS (Allen, 2011), Sahana (Careem et al., 2006), OpenIR (Ducao, 2013), and CrisisTracker (Rogstadius et al., 2013), provide support for mitigation and crisis preparedness. These tools pivot around the crowd for achieving great humanistic and environmental causes while leveraging the strength of geographically dispersed collaboration.

However, despite the growth of several initiatives and digital platforms designated to facilitate crowd intervention in emergency response, these initiatives are primarily based on individual contributions, without taking advantage of team dynamics that can arise among the crowd participants. This lack of communication, either due to team conflict (Yeo et al., 2018), or unfitness of the tools (Dilmaghani and Rao, 2006), makes crowdsourcing efforts less efficient, which often fail to address the event at hand, either as standalone initiatives or as supporting capacity to expert emergency management (Heath and Palenchar, 2000). Beyond the subject of crowdsourcing for emergency response, other team categories are also relevant to our research on *ad-hoc* crowd team formation. Action teams, rapid response teams, and citizen science, to name a few, are groups formed through the crowd and behave similarly to *ad-hoc* teams. Similar entities could benefit from system improvements addressing better team formation and communication strategies adopted from a better understanding of team dynamics in stressful situations. In the following subsection, we elaborate on existing—albeit early—efforts that seek to involve the crowd in formations and groups.

#### 2.2.2. Crowd Cooperation for Emergency Response

Aside from individual crowd contributions, a few studies have looked into facilitating communication among crowd members to respond to and manage unexpected events. Providing people with communication channels can help them gain a broader view of the event they need to deal with (Perez and Zeadally, 2019), and better coordinate their efforts (Martella et al., 2017). Song et al. (2020) analyzed a total of twelve international case studies of crowdsourcing and natural disaster governance. They denote that, across all of these instances, the crowd manifested (at least at some level in their response mechanisms) self-organizing properties that lead its individuals to form collaborative ties spontaneously. It suggests that the multi-directional relationship between the crowdsourcing platforms, the initiators, and the contractors, while not strictly guided, triggers the formation of functional teams that act as active response units. Under this instance, the crowd forms *ad-hoc* groups as the emerging outcome of community disaster resilience (Song et al., 2020). As long as collaboration is advantageous in emergency response and time management remains vital in real-life crises, boosting the efficacy of crowd participation starting from the level of team formation can get teams closer to their desired outcomes.

Many combinations of individual traits add up as building blocks for the entire social entity that is the team. Assuming that the single characteristic is, at least in principle, an optimal fit for the task, the way it interacts with the rest of the teammates' features is equally relevant. Personality clashes are present in virtual team interactions just as in traditional face-to-face cases. Following Van de Ven et al. (1976) definition of teams as “groups becoming more effective over time,” Salehi et al.'s (2017) work on stable crowd teams recognizes familiarity as the utmost important factor that enhances team performance. However, familiarity is a variable that cannot always be factored in when teaming up with individuals part of a virtual crowd, who are often sporadic contributors. Therefore, while familiarity in crowd teams has its tangible benefits (Salehi et al., 2017) for more stable tasks (like creative ones), relying on team familiarity to form effective crowd teams is not always feasible for short-lived, unpredictable, and mutable tasks.

For relatively short-lived assignments, the distribution of personality types matters more for the success and the establishment of trust in crowd teams than the pervasiveness of one specific type. Lykourentzou et al.'s (2016) work on crowd teams shows that balancing personality traits not only leads to significantly better performance on collaborative tasks but also reduces conflict and heightens the levels of satisfaction and acceptance. Holistically, when considering the impact of personality distribution in crowd teams, aspects other than personality traits play an often overlooked yet fundamental role. As Lykourentzou et al.'s (2016) noted: *test Personality could also be examined with regards to task type. For example, competitive tasks (like ideation contests among competing crowd teams) may amplify clashes within imbalanced teams, more than collaborative tasks*.” We aim to uncover the relevance of personality, communication, and other factors in a virtual emergency response task. Unlike other studies (Floch et al., 2012; Vivacqua and Borges, 2012; Ernst et al., 2017) evaluating crowd emergency response as a collective and self-organized effort, we propose a team-specific approach to the formation of crowd emergency units that strongly connects with theories and models of teams composition, and assembly and team science (National Research Council, 2015).

Closing, most crowdsourced initiatives for high-stake, high-pressure tasks rely on individual contributions. Few works use some form of teamwork to coordinate crowd participants' efforts spontaneously and not according to a systematic approach or criteria. The formation of crowd emergency teams according to a set of characteristics with known expected effects could help these teams experience less interpersonal conflicts, establish team cohesion faster, and increase the teams' chances of success. In this work, we systematize online team formation for high-pressure tasks. We closely investigate the effects of personality and communication patterns, contributing to such teams' success and helping harness the crowd's potential better in emergency response.

## 3. Study Design

Many factors may impact whether teams collaborate well and achieve their goals in an emergency response task. These include the demographics and personality of team members (both at the level of individuals and aggregated over the team), and the communication patterns used. This study explored which factors matter for team success and perceptions of collaboration quality. Given the many factors and output measures considered, the study was exploratory in nature, with the aim to gain initial insights into what matters and in which way, to be tested further in follow on studies.

### 3.1. Sample

120 Amazon Mechanical Turk workers (41 female, 78 male, 1 prefer not to say) participated. The task duration was approximately 20 min. Most participants were of U.S. (67 users) and Indian nationalities (51 users), one participant was Irish and another one was British. The majority had College (87) or Postgraduate degrees (15), while some had either some college education (9) or High School (9). Most were between 30 and 49 years of age. For an overview of the demographic data of the sample see **Table 7**.

### 3.2. Compensation

The participants received a base reward of $3, and a bonus reward of $3 if the challenge was completed successfully. The base pay was based on current fair crowd work compensation practices, whereas the bonus pay matched the base pay to double the reward for those teams that defused the bomb on time. The payment was weighted against the hourly rate or AMT workers as reported in Hara et al. (2018). In selecting the payment amount, we took into account three considerations from the literature (Olson and Kellogg, 2014; Lykourentzou et al., 2016). First, the payment had to conform to the community standards of the crowdsourcing platform so as not to bias the quality through workers who would accept low wages or workers who would only choose the task purely for its high compensation. Second, this payment had to cover the task duration. Thirdly, it took into account the demographics of the target worker population (minimum wage).

We recruited through the Amazon Mechanical Turk (AMT) Human Intelligent Task (HIT) platform. AMT was chosen for its breadth of crowd workers and its abundant labor availability, which is estimated to be no less than 2K workers at any given time, and over 100K workers overall (Difallah et al., 2018)^3^. No pre-selection was required to participate in the task. We intended to attract a large variety of participants, regardless of differences in background. The absence of pre-selection criteria may have influenced participants' written English, a limitation discussed in Section 6.2.3. Finally, the HIT itself contained information about the reward, the duration of the task, and a short description of the cooperative game.

### 3.3. Task Design and Setting

Although the task was artificial it was designed as an analog setting enacting the key characteristics of the high-demand, high-pressure environments that we are interested in. These include:

1. **Simulated element of physical danger**. The consequence of the team failing to navigate the maze is a bomb exploding. Although participants were aware that they are playing a game, the element of physical danger, even an enacted one, alters their perception, with possible effects on the way they process information, coordinate their efforts, and discuss (Kamphuis et al., 2011).
2. **Pre-determined team roles**. The presence of these roles enables stable and predictable group interactions (McMichael et al., 1999) instead of relying upon the slower and autonomous differentiation of team roles (Belbin, 2012), which cannot always happen in circumstances of emergency. Predefined role-playing exercised control over one's limited access to information, which symbolizes the relationship between an overseeing entity (in our case, the Lead Expert) and an operative agent (in our case, the Defuser). Furthermore, similar to real-life action teams, team membership symbolizes work shifts (Zijlstra et al., 2012). It represents the random assignment of roles on a first-come-first-served basis. Similar to emergency response teams, this approach creates teams with little time to explore personal similarities and differences or to go through classical team development processes (Tuckman and Jensen, 1977; Lacoursiere, 1980).
3. **Stress and increased consequences of failure**. The novelty of the task, alongside its short duration, positions the crowd participants in a situation similar to emergency management scenarios. Here, the users need to act decisively within tight time schedules, often only with access to incomplete or difficult to decode information (Carver and Turoff, 2007). It means that the participants (a) absorb information rapidly, (b) judge by doing, (c) decide on the spot, (d) deal with the event with little preparation. Users are aware that their actions, if wrong, will cost them (and their teammate) reasonably significant retribution (in this case monetary) (Driskell et al., 2018). The combination of elements, namely: high-stake, time-constrained, fractional information, and role inter-dependency, makes this particular task a reasonably stressful one. More so, the original game “Keep Talking Nobody Explodes” has been utilized as a tool by past research to assess the effects of realistic stress on behavioral and physiological responses of participants (Sabo and Rajčáni, 2017; Lee and Jung, 2020). These studies confirm that controlled environments of this sort can correctly reproduce similar stress levels of more realistic scenarios, thus inducing stimulus-response events—such as temporary homeostatic changes and speech variations— that signal increased stress.

**Figure 1:**
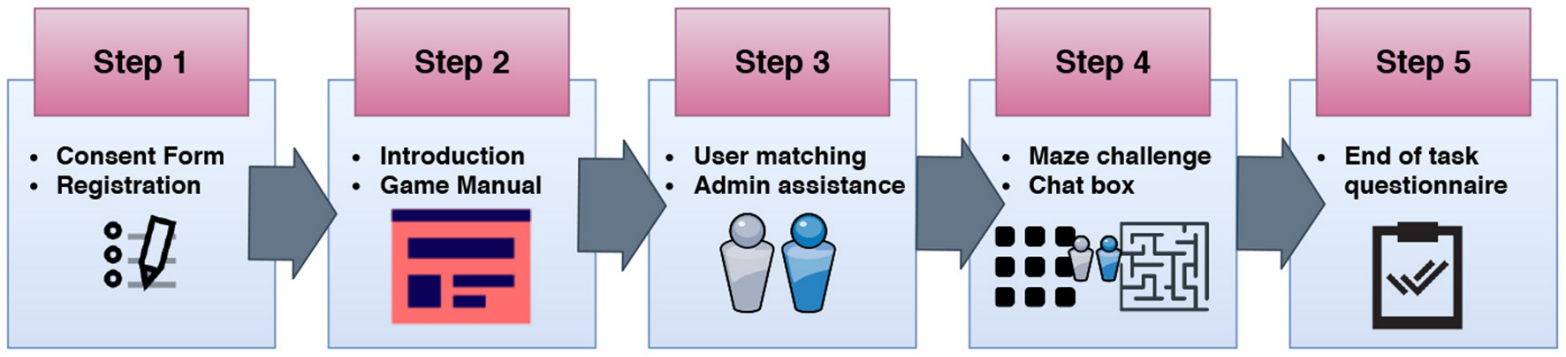
System overview with the five steps of the study design. After registration, users arrive at an introductory page with relevant information about the task, and then they are matched in dyads on a first-in-first-out basis. Each team then proceeds to their dedicated virtual room where they cooperate to defuse the bomb in the maze within a given time frame. Finally, they fill out a questionnaire about their abilities and perceived collaboration quality.

To support the task setting, we designed a custom-made web system. The system pipeline, illustrated in Figure 1, was designed according to the following steps:

**Step 1: Consent form and registration**. Participants registered with a username, AMT IDs (unique identifier needed to reward them at the end of the task), demographic information (gender, age, nationality, and education level), and Big-Five personality traits (**Table 3**). By registering, the participants agreed with the terms of service and gave their informed consent.

**Step 2: Introduction and game instructions**. After logging in, the “dangerous and challenging world of bomb defusing” (Knuth, 2021), the introductory page offered example screenshots of the two roles, instructions about the gameplay, plus information about the countdown and the end-of-task survey. The short info gave participants a broad idea of the task and focused on the platform functionalities (e.g., chat, game console, manual instructions, etc.).

**Step 3: User matching and admin assistance**. Participants entered the waiting room (i.e., matchmaking room) and were personally greeted by the system administrator while waiting for their teammates to join. If no other participants were present, they waited until a match would become available. The administrator also served as moderator and user support. The system allocated participants to teams in a first-in-first-out (FIFO) manner. As soon as two participants were present in the matchmaking room, they were placed together and asked to proceed to the main task (after first answering any questions they may have had).

**Figure 2:**
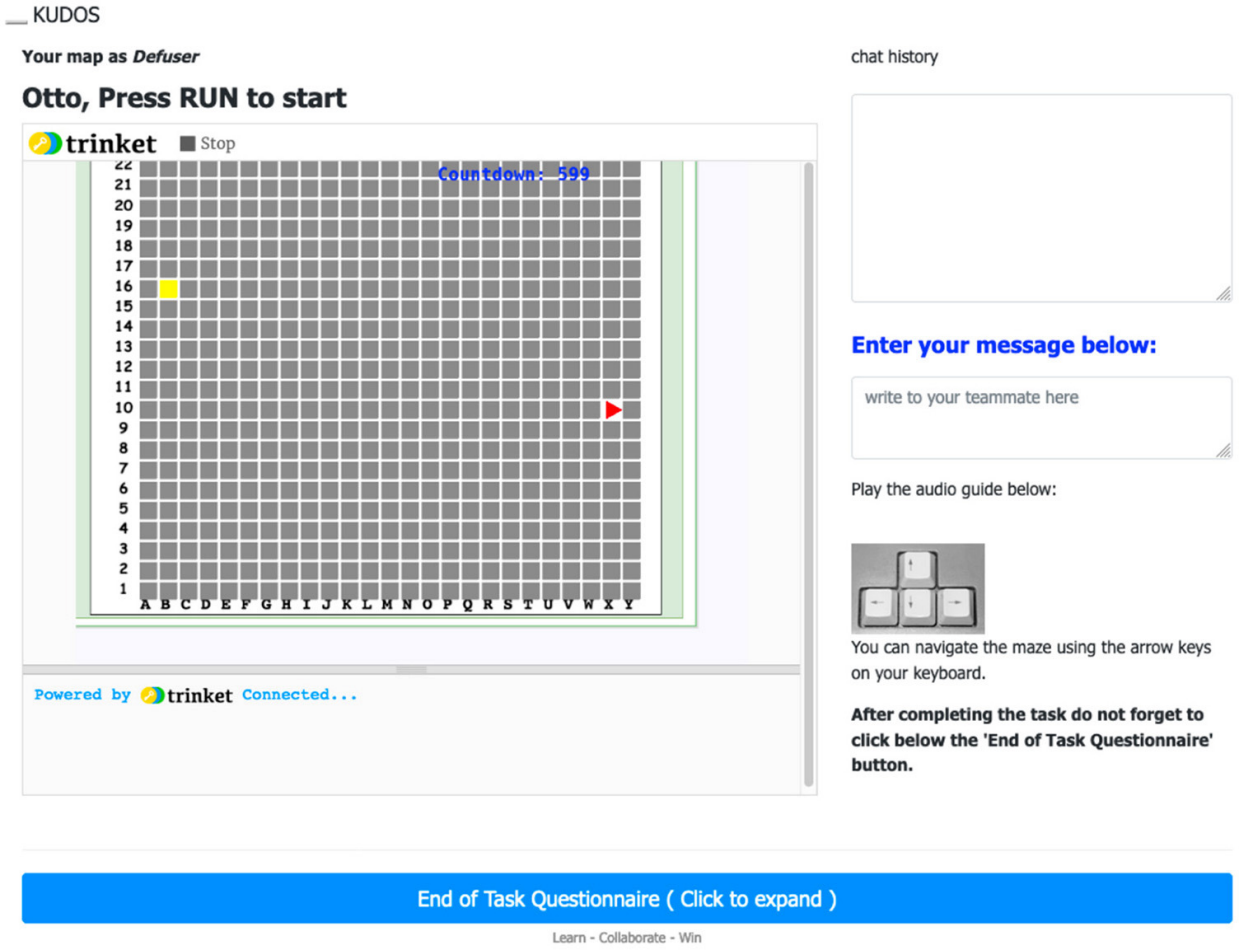
Defuser's view of the maze. The maze did not indicate the path to the bomb (red triangle), nor the walls. The participant was prompted to get directions from the Lead Expert through a chatbox (top-right of the screen).

**Figure 3:**
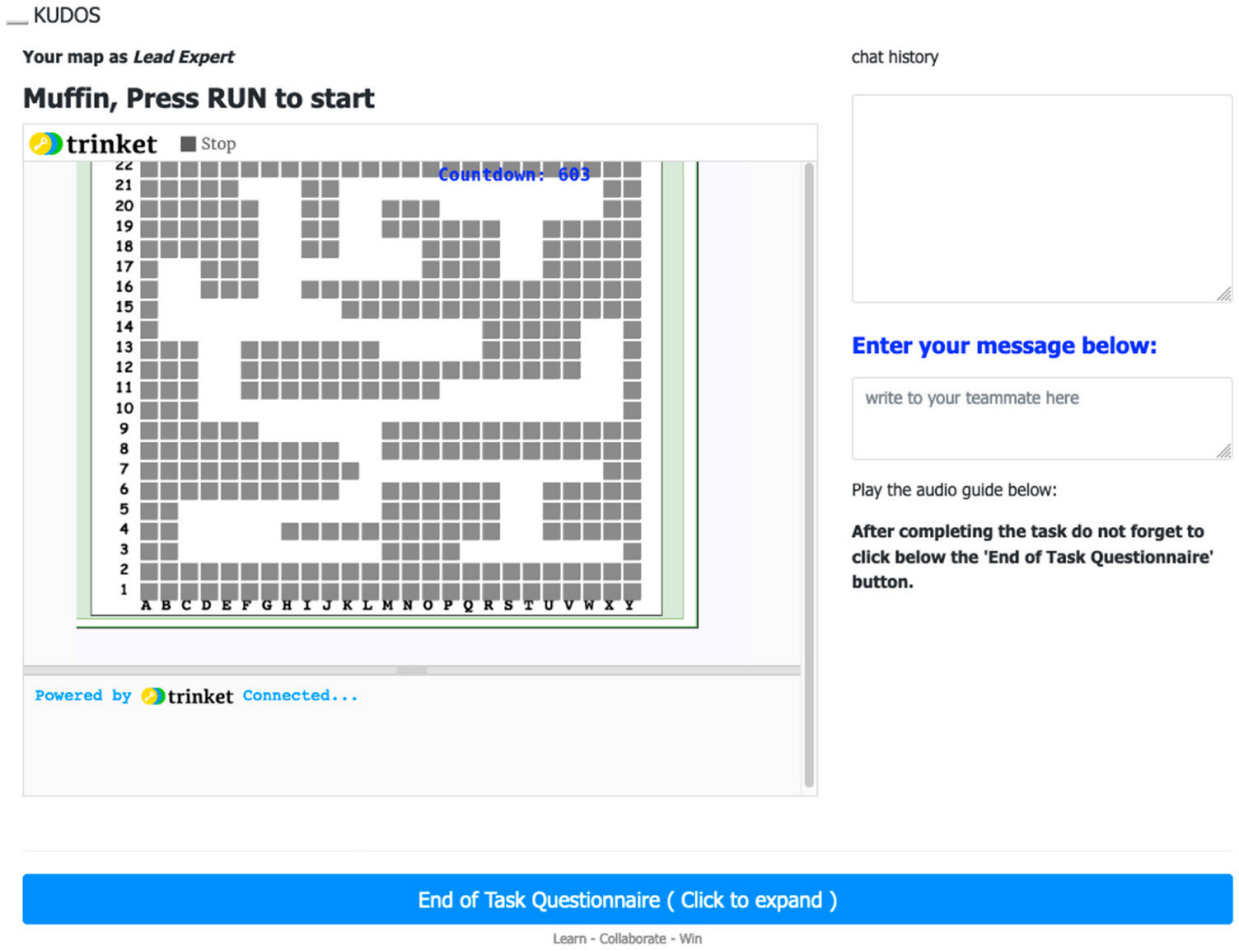
Lead Expert's view of the maze. The participant could see the map, but did not know where the bomb and the Defuser were placed in the map.

**Step 4: Maze challenge and chat box**. After matching, participants joined a private virtual room where they could see the maze game and chat to communicate with one another. Figure 2 shows what the Defuser saw. On the left-hand side, the Defuser saw a blind maze with their position (yellow square) and the bomb (red triangle). They could not see the walls as only the Lead Expert saw them. On the right-hand side, the Defuser saw the chatbox and, below it, a reminder to use the arrow keys to navigate the maze. Upon finishing the task, the blue bar at the bottom of the screen would take them to the final questionnaire. Figure 3 shows what the Lead Expert saw. The Lead Expert's view of the maze differed from that of the Defuser: they saw only the walls of the maze (gray squares) and the path to the bomb (white sections). The Lead Expert could neither see the Defuser in the maze nor the bomb. Both the Lead Expert and Defuser could see the same countdown and Cartesian coordinates of the maze, as well as the chatbox and the link to the final questionnaire.

The Maze module was inspired by the video game “Keep Talking Nobody Explodes” (Knuth, 2021). It consisted of a 25 x 25 grid of squares with one square containing a yellow element (the position of the Defuser), one square containing a red triangle (the position of the bomb), and walls. Neither of the two players had access to all the information of the maze; they needed to cooperate. The Defuser could move inside the maze, by means of the four arrow keys, but they did not know where the walls were. The Lead Expert had the map, but could not navigate the maze. The Defuser's role was to navigate the maze, with the help of the Lead expert, and defuse the bomb in time. Finally, a countdown timer was included, at the end of which the bomb exploded, unless it had been defused. The countdown started the moment both players entered the room. For this specific study, the timer was set to 400 s. After finishing the game, the participants received a validation code to submit to the AMT HIT for getting their base pay and bonus reward (for those teams that completed the challenge successfully). We deliberately excluded aspects of the original video game to reduce the number of variables and increase the controllability of the study environment. We wanted participants to focus on reaching the bomb on time without spreading themselves thin among the multi-modalities present in the original game (e.g., clues, strikes, wires, sequences, etc.). Besides, implementing most features of the original game would have added to the task complexity^4^. Hence, we did not include penalties for the Defuser colliding with a wall. The only penalty—and end of game—was determined by the time running out before reaching the bomb. Furthermore, to ensure task brevity, we considered the bomb defused as soon as the Defuser stepped inside its cell. The simplification of the game has some limitations discussed in Section 6.2.

**Step 5: End of task questionnaire**. Participants rated the perceived collaboration quality on multiple aspects (see below), and also their abilities.

## 4. Metrics

We grouped the multilevel approach into two distinct classes referring to input and output variables (Table 2 provides a summary of all variables, their type and range.). Here the input metrics serve as the independent variables and the output ones as dependent variables.

**Table 2:** Summary of variables.

		**Variable**	**Type**	**Range**
		Extraversion	Interval	2–10
		Agreeableness	Interval	2–10
	Personality^5^	Conscientiousness	Interval	2–10
		Emotional stability	Interval	2–10
		Openness to experience	Interval	2–10
		StDev	Ratio	0–5.66
	Team Personality (for each trait)	Min	Interval	2–10
		Max	Interval	2–10
		Mean	Interval	2–10
Input		Gender	Nominal	Male, Female, Other, not-disclosed
	Demographics	Age group	Ordinal	<20, 20–29, 30–39, 40–49, 50+
		Nationality^6^	Nominal	USA, India, UK, Ireland
		Education level	Ordinal	Less than High School, High School (HS), Some College (SC), College degree (Col), Postgraduate (PG)
	Communication patterns	Uncertainty, Action, Response, Planning, Factual, Non task-related	Ratio	≥0
		Chat length (# Words)	Ratio	≥0
		Chat total (# Posts)	Ratio	≥0
	Performance		Nominal	Won, Lost
		Performance	Ordinal	1–5
Output	Perceived collaboration quality	Cohesion Communication quality Balance Satisfaction	Ordinal Ordinal Ordinal Ordinal	1–5 1–5 0–2 0–2

### 4.1. Input Variables

#### 4.1.1. Big Five Personality Traits

To acquire a measure of the Big Five traits within the context of large-scale assessment under limited time and resources, we used the Big Five Inventory-10 (BFI-10) (Rammstedt and John, 2007). The inventory consists of ten questions (see Table 3). Derived from the shortening of its lengthier predecessor (the Big Five Inventory (BFI-44) Rammstedt and John, 2007), it focuses on the psychometric characteristics of the BFI-44's most representative items and reduces each Big Five dimension to 2 BFI items. The BFI-10 measures the personality traits of Extraversion, Agreeableness, Conscientiousness, Emotional Stability (Neuroticism), and Openness to experience (Rammstedt and John, 2007)^8^. For each trait, the BFI-10 score is calculated as the total score of the two statements associated with that trait, after reversing the score of some statements (see mapping of statements to traits and which statements' scores are reversed in Table 3)^9^.

**Table 3:** BFI-10 instrument used, and its scoring: the trait for which each item was used and whether it was reverse scored (R)^7^.

**I see myself as someone who …**	**Disagree**	**Disagree**	**Neither agree**	**Agree**	**A gree**	**Trait**	**Reverted**
	**strongly**	**a little**	**nor disagree**	**a little**	**strongly**		
1. … is reserved	(1)	(2)	(3)	(4)	(5)	Extraversion	R
2. … is generally trusting	(1)	(2)	(3)	(4)	(5)	Agreeableness	
3. … tends to be lazy	(1)	(2)	(3)	(4)	(5)	Conscientiousness	R
4. … is relaxed, handles stress well	(1)	(2)	(3)	(4)	(5)	Neuroticism	R
5. … has few artistic interests	(1)	(2)	(3)	(4)	(5)	Openness to Experience	R
6. … is outgoing, sociable	(1)	(2)	(3)	(4)	(5)	Extraversion	
7. … tends to find faults with others	(1)	(2)	(3)	(4)	(5)	Agreeableness	R
8. … does a thorough job	(1)	(2)	(3)	(4)	(5)	Conscientiousness	
9. … gets nervous easily	(1)	(2)	(3)	(4)	(5)	Neuroticism	
10. .. has an active imagination	(1)	(2)	(3)	(4)	(5)	Openness to Experience	

#### 4.1.2. Personality Traits of Groups

There is no straightforward process for aggregating metrics such as personality traits for groups. However, the group recommender community has dealt with a similar issue namely the aggregation of group members preferences (Masthoff, 2004) and uses aggregation strategies from Social Choice Theory (Sen, 1986). Senot et al. (2010) distinguishes between (1) majority-based strategies that use the most popular values, (2) consensus-based strategies that consider the profiles of all group members, and (3) borderline strategies that only consider a subset. In our case, majority strategies do not apply given a group size of two. Of the consensus-based strategies, we use Average (which is also the most popular strategy in Group Recommender research). Of the borderline strategies, we use Minimum and Maximum^10^^,^^11^. Minimum is used as one may expect that team performance is strongly affected by the weakest member in the team, in line with the popular saying “a chain is as strong as its weakest link”. Maximum is used as one may also expect that a strong member could make up for the weakness in another member (e.g., if one person is highly conscientious, they may entice the team to get the work done in time), particularly when the team is small. Finally, we used Standard Deviation (in line with the Cohesion metric introduced by Odo et al., 2019b), as the literature indicates the impact of diversity within teams^12^.

#### 4.1.3. Demographics

Participants provided information about their gender, age group, nationality, and educational background. Socio-demographic measures identify characteristics that often influence the respondent's opinions that could condition one's behavior, culture, and experiences (Lavrakas, 2008). These socio-demographic factors provide further insight into the composition of teams, and what other characteristics—aside from personality traits—influence the collaboration. These socio-demographic factors that make someone distinct can turn into assets for group work. Therefore, by being aware of those characteristics, organizations and hiring bodies can better assemble and coordinate geographically dispersed teams (Muethel et al., 2012).

Multiple studies (Ruef et al., 2003; O'Leary and Mortensen, 2010; Akman et al., 2011) have identified various aspects of the teammates' social backgrounds and demographic characteristics that condition teamwork. For example, members of similar demographic profiles have greater chances to kindle stronger affinity ties (Ruef et al., 2003). Other demographic differences, such as race, sex, age, and nationality, have also been found (Martins and Shalley, 2011) to affect the collective creativity of virtual teams. Age differences condition the creative processes of teams and intensify differences in technical experience (Martins and Shalley, 2011). Differences in nationality have a negative effect by interacting—however indirectly—with the technical experience of the teammates (Martins and Shalley, 2011).

#### 4.1.4. Communication Patterns

The methodology by Bowers et al. (1998) introduced a new approach to communication analysis prompted by a prior research gap in metrics that missed to analyze the more fine-grained interaction patterns other than simple frequency counts of words. They proposed the implementation of the categories of: (a) **uncertainty** statements, which included direct and indirect questions; (b) **action** statements, which required a particular member to perform a specific action; (c) **acknowledgments**, which were one-bit statements following uncertainty of action statements, such as “yes,” “no,” “roger”; (d) **responses**, which differed from acknowledgments only in that they conveyed more than one bit of information; (e) **planning** statements; (f) **factual** statements, which verbalized readily observable realities of the environment; and (g) **non task-related** statements. These categories quantified the performance of crews during simulated flight tasks, which improved the make-up of communication sequences analysis.

Based on Bowers et al. (1998) contribution, Davaslioglu et al. (2019) developed the Collective Allostatic Load Measurers system (CALM), which collected, aggregated, and analyzed data from individuals to make assessments on team situation awareness, performance, and resilience. The study used the virtual-reality game “Keep Talking Nobody Explodes” that we too used as inspiration for our experiments. Davaslioglu et al.'s (2019) study demonstrated that some teams exhibited patterns of communication, namely, action-response, uncertainty-response-action, and factual-uncertainty-response-action while working together under high-stress conditions. Acknowledgment statements, for instance, were seen to predominate more amongst high-performing teams, while low-performing teams had higher portions of non-task-related-statements. Similar studies on team communication analysis (Pfaff, 2012; Zijlstra et al., 2012) have identified patterns of communication. Given the proximity of our methodology to the studies of Bowers et al. (1998) and Davaslioglu et al. (2019), we implemented the same communication classes as they did. These communication patterns, or categories, are the following:

- **Uncertainty**. Uncertainty statements comprise questions (either direct or indirect) about the task (e.g., “Where are you at?,” “Where is the bomb?”).
- **Action**. Action statements indicate that one or both of the team members are taking action inside the game, or they are a direction to take action (e.g., “Move two steps down, then one right.” “I am moving to position *x*,” or “Go up for three blocks, then turn right”).
- **Responses**. Response statements can accompany either uncertainty or action statements and suggest that a communication, or feedback loop (e.g., “yes,” “no”), is ongoing.
- **Planning**. Planning statements that give the users a feeling that they are working together toward achieving a common goal. Planning statements can indicate the user's ability to reassess the situation, clarify the work, or plan the next actions.
- **Factual**. Factual statements are situational and describe the reality, for instance, by giving cues about how the maze looks like from the viewpoint of the Lead Expert, or at which coordinates the bomb is located.
- **Non task-related**. Non-task-related statements are parts of the chats that are categorized as non-related when they do not contribute to the achievement of the goal (e.g., “What is the weather like?”).

Table 4 illustrates an extract of the annotated chat between the Lead Expert and the Defuser. The patterns were labeled for each participant's text entry and annotated by two independent evaluators. The inter-rater agreement of the annotation was sufficiently high to be utilized in the study (Cohen's κ = 0.998, *p* = 0.000). In addition to counting how often each communication category was used, we also counted the total number of posts made (chat total) and the number of words used (chat length).

**Table 4:** Example of an annotated chat sequence between a Lead Expert and a Defuser.

**Text**	**Annotation**	**Role**
Okay?	Response	Defuser
Got it?	Response	Lead Expert
I don't see bomb on my screen, do you know?	Uncertainty	Defuser
I'm the yellow square	Factual	Defuser
czzan't see bombs	Factual	Lead Expert
where r u?	Uncertainty	Lead Expert
16C	Factual	Defuser
go to 12x	Action	Lead Expert
where should I go?	Uncertainty	Defuser
One step at a time	Planning	Lead Expert
As a lead expert, I request you to guide me	Planning	Lead Expert
Both of us should use the code	Planning	Lead Expert
even I can't see the bomb	Factual	Lead Expert
there is a triangle on L3	Factual	Defuser
ok	Response	Lead Expert
wait	Action	Lead Expert
can you move? Take turns moving maybe?	Uncertainty	Defuser
follow my steps	Action	Lead Expert
How is your family members?	Non-Related	Defuser

### 4.2. Output Variables

#### 4.2.1. Team Performance

Ancona and Caldwell's (1992) definition of team performance is the extent to which a team can meet its output targets (e.g., quality, functionality, and reliability of outputs), the expectations of its members, or it's cost and time goals (Ancona and Caldwell, 1992). For this study, the team performance metric consisted of the binary mapping of the task outcome (winning/losing). The team performance metric has been used as a dependent variable in our functional analysis of the collaboration to illustrate the role of the input factors (personality traits and communication patterns) and allow us to evaluate the constitution of those teams.

#### 4.2.2. Perceived Collaboration Quality

To measure perceived collaboration quality, we use five metrics of team dynamics, which evaluated the participants' perceptions of their teams.

##### 4.2.2.1. Perceived Performance

The perceived performance metric addresses the question “*How well, in your opinion, did your team perform?.”* It was measured on a five-point Likert-scale from “*Very poorly”* (1) to “*Very well”* (5) The perceived performance variable defines the subjective layer of teamwork capability at the given task. The notion has been conceptualized as a multilevel process arising as the teammate engages in their individual and team-level task-work and teamwork processes (Kozlowski and Klein, 2000).

##### 4.2.2.2. Perceived Cohesion

The perceived cohesion metric addresses the question: “*How cohesive was your team?,”* measured using a similar 5-point Likert-scale. Perceived team cohesion, as a fringe term covering social relations, task relations, perceived unity, and emotions (Beal et al., 2003), contributes to our understanding of the emotional dimension of the teams, which is a rather subtle corollary facet of teamwork alongside other subjective measures. The study proposes that group members' perceptions of their cohesion to a particular group are essential in the sense of belonging and feelings of morale (Bollen and Hoyle, 1990). More so, the meta-analysis by Beal et al. (2003) clarifying the construct relation between this particular subjective metric and team performance has denoted a high correlation between these factors across several studies on teams. This work has further established the importance of cohesion (including the subjective measurement) in team performance.

##### 4.2.2.3. Perceived Communication Quality

The perceived communication quality metric addresses the question: “*How well did your team communicate?,”* measured using a similar 5-point Likert-scale. Collecting the perception of the communication quality can help us encode important information about the participant's beliefs toward how a team should function. It can also help disclose the way that the respective individuals engage in communication with the other team members and the way they perceive the communication ties (Cook et al., 2020). Differences in perception might uncover discrepancies between teammates' viewpoints that can lead to the establishment of complex team interventions that intervene at multiple levels of the team formation and interaction processes (Wauben et al., 2011).

##### 4.2.2.4. Perceived Balance

The metric addresses the question: “*Did both members of your team contribute equally in your opinion?”* measured using a 3-point Likert-scale. The variable links with the staging of roles and responsibilities within a team, including how they distribute between teammates and the ways they get carried out against the team's objectives (van de Water et al., 2008). To understand the relevance of the metric within the present study design, remember how entirely different the two roles are and how diametrically determinant they can contribute to teamwork. The top-down allocation of roles was, by itself, not a sufficient guarantee that the teammates' behavior aligned with the given role. By assessing the aspect of perceived balance, through the lenses of the teammates, we could better understand what the participants, and whether it was indeed a balanced act or whether a role was considered more demanding and accountable for the outcome than the other.

##### 4.2.2.5. Satisfaction

The metric addressed the question: “*Would you play with the same teammate again?”* measured using a 3-point Likert-scale. Satisfaction helps predict whether a combination of participants will more likely prefer to work with similar teammates in the future.

## 5. Results

We divide our results into two themes: 1. performance, and 2. perceived collaboration quality.

1. **Team performance:**
  - **Section 5.1** analyzes the effect of personality at team level ^13^, comparing winning to losing teams to see if there may be a relationship between personality and performance. It reports the results of a Mann-Whitney U test and perform a regression to investigate the relationship between team traits and the likelihood of a team winning.
  - **Section 5.2** analyzes the communication patterns using a one-way ANOVA to compare winners and losers, but also to compare the differences in behavior between the team roles.
  - **Section 5.3** evaluates the impact on team performance of the participants' socio-demographic characteristics, using Chi-square tests and regression analysis.
2. **Perceived collaboration quality:**
  - **Section 5.4** assesses the relationship between personality traits and perception of collaboration quality, using correlation analysis for the individual traits.
  - **Section 5.5** assesses the relationship between personality traits and perception of collaboration quality, using correlation analysis for the team traits.
  - **Section 5.6** examines whether individual demographic characteristics played any role in people's perception of their collaboration, using one-way ANOVAs.
  - **Section 5.7** analyzes the relationship between the communication patterns and the collaboration quality metrics, also considering the roles of the Defuser and Lead Expert, using correlation analysis.

Given the many factors considered (e.g., considering 5 personality traits with 4 different aggregation metrics for team personality already results in 20 factors) and the many outcome measures, many statistical tests were performed. This may lead to Type I errors. Using Bonferroni corrections^14^ to avoid Experiment wide Type I errors would reduce the power of the statistical tests to such an extent that Type II errors would be highly likely and few insights would be gained^15^. We have therefore not applied such corrections (except in *post-hoc* pairwise comparisons). The study is exploratory in nature, and the statistical results presented provide initial insights that lead to hypotheses for follow-on studies.

### 5.1. Impact of Personality on Team Performance: Minimum Openness May Matter

Since there is no universally accepted way of aggregating team member personality traits into team personality traits, we used multiple, namely the average, minimum, maximum, and standard deviation. Each of these metrics was examined in isolation, as they are not independent. Table 5 shows the mean (and standard deviation) of these four metrics for the winning and the losing teams. Minimum Openness was significantly better in winning teams (Mann-Whitney *U* = 485, *p =* 0.02). There were no other significant results^16^.

**Table 5:** Mean (Stdev) of standard deviation, average, minimum, and maximum for personality traits for winning and losing teams.

		**Openness**	**Conscientiousness**	**Extraversion**	**Agreeableness**	**Neuroticism**
	StDev	1.06 (0.68)	1.41 (1.46)	1.15 (1.36)	1.50 (1.59)	1.10 (1.00)
Winning	Average	8.13 (1.51)	7.75 (1.53)	5.75 (2.32)	6.94 (1.53)	4.22 (2.33)
Teams	Min	7.38 (1.71)	6.75 (2.24)	4.94 (2.65)	5.88 (2.25)	3.44 (2.42)
	Max	8.87 (1.46)	8.75 (1.34)	6.56 (2.37)	8.00 (1.46)	5.00 (2.45)
	StDev	1.72 (1.36)	1.11 (1.32)	1.66 (1.52)	1.46 (1.25)	1.96 (1.88)
Losing	Average	7.26 (1.60)	8.24 (1.41)	5.01 (1.55)	6.40 (1.26)	3.82 (1.74)
Teams	Min	6.05 (2.22)	7.45 (1.95)	3.84 (1.80)	5.36 (1.79)	2.43 (1.37)
	Max	8.48 (1.42)	9.02 (1.39)	6.18 (1.97)	7.43 (1.25)	5.20 (2.78)

A binary logistic regression with the minimum metric^17^ considered the effects of the teams personality on the likelihood of winning^18^. Given only 16 out of 60 teams won, the basic model only uses a constant with an accuracy of 73.3% (obtained by always predicting the team will lose). The logistic regression model was statistically significant, χ^2^_(6)_ = 13.60, *p* = 0.034. The model explained 30% (Nagelkerke R^2^) of the variance in winning and correctly classified 77% of cases, including 38% of wins. Increasing minimum Openness and minimum Neuroticism were associated with an increased likelihood of winning [Openness: Exp(B) = 1.52, Wald = 4.61, *p* = 0.032; Neuroticism: Exp(B) = 1.58, Wald = 4.20, *p* = 0.041].

Our results indicate that in this kind of task (high-pressure, high-demand), minimum Openness to experience seems the most important factor among the Big-5 traits in helping the team to effectively manage the *ad-hoc* collaboration to find a winning solution within a limited time. This means that a crowdsourced, *ad-hoc*, and remote emergency response team will likely be more successful at executing a time-bounded novel task if both collaborators share high levels (minimum) of Openness to experience. The minimum level of this trait indicates that teams with individuals with low Openness are expected to hamper the collaboration regardless of whether the counterpart has very high levels of Openness and this is reasonably determined by the interdependence between roles.

### 5.2. Impact of Communication Patterns on Team Performance: Action and Response Help Teams Win

Table 6 shows the number of posts per chat category for winners and losers, for winning and losing teams, and for Defusers and Lead Experts. As the role likely affects how participants communicate, we analyzed the communication pattern usage data at the individual level, with an output variable whether these people belonged to winning or losing teams. We analyzed the six chat categories (Uncertainty, Action, Response, Planning, Factual, Non-related), the chat length (in words) and the total number of chat posts between winners and losers using a one-way ANOVA. Winners used significantly more *Action* and *Response* statements [*F*_*action*_(1,118) = 4.426, *p =* 0.038, *F*_*response*_(1,118) = 4.983, *p* = 0.027].

**Table 6:** Mean (Stdev) of number of times chat categories were used by winners and losers, by winning and losing teams, by Defusers and Lead Experts, and total usage by each.

	**Uncertainty**	**Action**	**Response**	**Planning**	**Factual**	**Non-related**	**Total**
Winners	2.03 (3.10)	2.91 (4.85)	3.41 (3.77)	0.28 (0.58)	2.34 (2.89)	0.03 (0.18)	11.00 (11.15)
Losers	1.94 (2.30)	1.45 (2.60)	2.14 (2.29)	0.17 (0.49)	2.13 (2.49)	0.52 (2.82)	6.71 (11.00)
Winning teams	4.06 (4.71)	5.81 (6.66)	6.81 (7.08)	0.56 (1.09)	4.69 (4.47)	0.06 (0.25)	22.00 (20.41)
Losing teams	3.89 (3.27)	2.91 (3.67)	4.27 (4.01)	0.34 (0.77)	4.25 (4.21)	1.05 (4.08)	16.70 (11.55)
Defusers	1.62 (2.29)	0.72 (1.29)	2.32 (2.70)	0.27 (0.58)	2.72 (2.87)	0.07 (0.41)	7.70 (6.88)
Lead experts	2.32 (2.72)	2.97 (4.35)	2.63 (2.92)	0.13 (0.43)	1.65 (2.18)	0.72 (3.39)	10.42 (9.14)

A binary logistic regression model to predict whether a participant would win or lose was statistically significant [χ ^2^_(7)_ = 14.86, *p* = 0.038]. The model explained 17% (Nagelkerke R^2^) of the variance in winning and correctly classified 78% of cases (25% wins). Increasing the *Action* and *Response* categories was associated with an increased likelihood of winning [Exp(B) = 1.28, Wald = 5.35, *p* = 0.021; Exp(B) = 1.21, Wald = 3.92, *p* = 0.048, respectively]. Increasing the chat length was associated with a decreased likelihood of winning [Exp(B) = 0.97, Wald = 4.04, *p* = 0.044]. These results seem to indicate that participants who gave feedback to one another and focused on discussing which action to take—rather than other types of communication—were able to finish the task and win the game. We also understand that the amount of chat is not a sufficient measure for success in online emergency response team settings since we could not find neither correlation nor causality between these variables.

Lead Experts used the Action category significantly more than Defusers [*F*_*action*_(1,118) = 14.736, *p* < 0.001] whilst Defusers used the Factual category significantly more [*F*_*factual*_ (1, 118) = 5.273, *p =* 0.023]. The Lead Experts are the ones with the map and would direct the Defusers to the appropriate path to defuse the bomb. Meanwhile, the Defusers may need to tell the Lead Experts where they are. There is a statistically significant difference in the chat categories, with Defusers on winning teams using a significantly higher proportion of Factual messages in their chat than those on losing teams (53 vs. 33%, *p =* 0.043) and a lower proportion of Uncertainty messages (8 vs. 22%, *p =* 0.041).

### 5.3. Impact of Socio-Demographic Characteristics on Performance

Table 7 shows the demographics of winners vs. losers, excluding cases with very low frequency^19^. Pearson Chi-square tests show a significant association between gender and winning [χ^2^_(1,*N* = 119)_ = 4.78, *p =* 0.029] and age and winning [χ^2^_(3, *N* = 120)_ = 8.09, *p =* 0.044]. Men were more likely to win. A binary logistic regression model to predict whether a participant would win or loose based on gender was statistically significant [χ ^2^_(1)_ = 5.12, *p* = 0.024]. However, it only explained 6% of the variance in winning and correctly classified 73.1% of cases only by always predicting losing. Being female was associated with a slightly decreased likelihood of winning [Exp(B) = –1.07, Wald = 4.53, *p* = 0.033]).

**Table 7:** Demographics overall and of winners vs. losers (excluding prefer not to say for gender and nationality) and also for teams that include the same or different genders and nationalities.

	**Gender**				**Nationality**				**Age**				**Education**			
	**Men**	**Women**	**Same**	**Differs**	**USA**	**India**	**Same**	**Differs**	**20–29**	**30–39**	**40–49**	**50+**	**HS**	**SC**	**Col**	**PG**
N	78	41	33	27	67	51	33	27	23	56	26	15	9	9	87	15
Winners	33%	15%	30%	22%	19%	35%	27%	26%	22%	36%	27%	0%	11%	33%	28%	27%
Losers	67%	85%	70%	78%	81%	65%	73%	74%	78%	64%	73%	100%	89%	67%	72%	73%

We also investigated whether adding gender to the model that uses personality to predict winning would improve the model. A binary logistic regression model to predict whether a participant would win or loose based on gender as well as team personality (in terms of minimum Openness and Neuroticism given the results from Section 5.1) was statistically significant [χ^2^_(3)_ = 27.97, *p* < 0.001]. The model explained 31% (Nagelkerke R^2^) of the variance in winning and whilst correctly classifying 78.2% of cases. Being female was associated with a decreased likelihood of winning [Exp(B) = –1.31, Wald = 4.97, *p* = 0.026]. Similar to our earlier results, increases in minimum Openness and Neuroticism were associated with an increased likelihood of winning [Exp(B) = 0.47, Wald = 11.92, *p* = 0.001; Exp(B) = 0.52, Wald = 11.94, *p* = 0.001, respectively]. A similar model without Gender explained only 25% of the variance in winning, and reduced correct classification to 76.5%. Thus, gender mattered but less than personality. When age, nationality or education are added to the binary logistic model instead of gender, they are not significant.

### 5.4. Impact of Individuals Personality Traits on Perceived Collaboration Quality: Agreeableness May Be Helpful to Cope With Losing

Unfortunately, only 44 out of 120 participants (23 Lead Experts and 21 Defusers) completed the survey at the end of the task, concerning their perception of their team's Cohesion, Performance, Communication, Balance, and Satisfaction. All perceived collaboration metrics were positively correlated (see Table 8), overall and for winners. In contrast, for losers the correlations with Satisfaction were not significant (see Table 8), and Performance and Balance were also not correlated. So, losers may not always have attributed the bad performance to a poor balance in the team, nor always have been unwilling to keep working with a person even though the collaboration was not going well (according to the other metrics and the fact they lost).

**Table 8:** Spearman correlations between perceived collaboration quality metrics, **p < 0.01, *p < 0.05.

		**Performance**	**Cohesion**	**Communication**	**Balance**	**Satisfaction**
	Performance	1	0.751**	0.593**	0.449**	0.525**
	Cohesion	0.751**	1	0.649**	0.528**	0.502**
All (*N* = 44)	Communication	0.593**	0.649**	1	0.506**	0.508**
	Balance	0.449**	0.528**	0.506**	1	0.389**
	Satisfaction	0.525**	0.502**	0.508**	0.398**	1
	Performance	1	0.732**	0.648**	0.486*	0.568**
	Cohesion	0.732**	1	0.725**	0.512*	0.579**
Winners (*N* = 24)	Communication	0.648**	0.725**	1	0.530**	0.646**
	Balance	0.486*	0.512*	0.530**	1	0.484*
	Satisfaction	0.568**	0.579**	0.646**	0.484*	1
	Performance	1	0.734**	0.523*	0.302	0.299
	Cohesion	0.734**	1	0.514*	0.419	0.319
Losers (*N* = 20)	Communication	0.523*	0.514*	1	0.470*	0.283
	Balance	0.302	0.419	0.470*	1	0.261
	Satisfaction	0.299	0.319	0.283	0.261	1

Agreeableness significantly correlated with perceived Performance, Cohesion, and Balance. Neuroticism significantly correlated with only Balance (see Table 9). Considering only winners, there were no significant correlations between the personality traits and any metric. In contrast, losers had a significantly positive correlation on Agreeableness with Performance, Cohesion, and Communication. Furthermore, losers had a significant negative correlation on Conscientiousness with Communication. Agreeableness may have helped people to see their loss in a more positive light, making them feel more positively about their teams performance, communication and cohesion^20^^,^^21^. We do not know whether being more conscientious made losers feel worse about their teams communication, or whether the team communication was influenced negatively by their Conscientiousness. The lack of a significant correlation for winners points toward the first explanation, with Conscientious people perhaps being more honest in assessing team communication quality.

**Table 9:** Correlations between perceived collaboration quality metrics and personality traits, **p < 0.01, *p < 0.05.

		**OPEN**	**CONS**	**EXTRO**	**AGR**	**NEUR**
	Performance	0.062	–0.187	0.044	0.434**	0.106
	Cohesion	0.050	–0.181	–0.088	0.319*	0.160
All (*N* = 44)	Communication	–0.111	–0.256	–0.217	0.221	0.159
	Balance	–0.029	–0.203	–0.196	0.317*	0.318*
	Satisfaction	–0.003	–0.035	–0.074	0.032	–0.031
	Performance	0.081	–0.099	0.064	0.289	–0.023
	Cohesion	0.053	–0.148	–0.006	0.241	0.013
Winners (*N* = 24)	Communication	–0.068	–0.098	–0.239	–0.074	0.044
	Balance	–0.319	–0.302	–0.345	0.354	0.285
	Satisfaction	–0.086	0.144	–0.009	–0.072	–0.098
	Performance	0.013	–0.336	0.017	0.761**	0.330
	Cohesion	0.021	–0.226	–0.162	0.456*	0.388
Losers (*N* = 20)	Communication	–0.178	–0.551*	–0.159	0.547*	0.397
	Balance	0.315	–0.053	0.004	0.338	0.361
	Satisfaction	0.025	–0.233	–0.112	0.242	0.050

### 5.5. Impact of the Teams Personality Traits on Perceived Collaboration Quality: The Positive Role of openness and Surprising Need for Conscientiousness Differences

We determined values for a teams perceived collaboration quality metrics by taking the average of its members, or only one member had provided their ratings by using that members ratings. Average and minimum Openness positively correlated with perceived performance^22^ in line with earlier findings that Openness had a positive impact on the likelihood of a team winning. Maximum Agreeableness positively correlated with perceived performance^23^, in line with our earlier observations regarding the impact of Agreeableness on individuals opinions.

The most interesting result is the significant positive correlation of all perceived quality metrics with Conscientiousness standard deviation^24^^,^^25^.

A lower Conscientiousness standard deviation correlated with negative team's feelings. In a dyad, the lowest Conscientiousness standard deviation is when two people work together who are very similar in Conscientiousness. For example, two highly conscientious people or two lowly conscientious people. Two lowly conscientious people working together may not result in a good collaboration. However, two highly conscientious people working together are likely to yield good performance. It seems that the best performance—from the team members' point-of-view—for this particular type of task comes from two people differing in Conscientiousness working together.

### 5.6. Impact of Socio-Demographic Characteristics on Perceived Collaboration Quality: No Significant Result

Tables 10, 11 show the perceived collaboration quality metrics for the different genders, age groups, nationalities, and education levels. One-way ANOVAs showed no significant effect of socio-demographic variables on perceived team performance, cohesion, communication, balance, and satisfaction^26^. The averages on all metrics except for balance were a bit higher for men (which would make sense given the men had more often won), but this was not statistically significant, which is not surprising given the high variance and the sample size.

**Table 10:** Mean (standard deviation) of collaboration quality metrics by gender and age, and also for teams that include the same or different genders.

**Collaboration**	**Gender**				**Age**			
	**Men (32)**	**Women (12)**	**Same (20)**	**Differs (14)**	**20–29 (11)**	**30–39 (25)**	**40–49 (6)**	**50+ (2)**
Performance	3.75 (1.27)	3.17 (1.53)	3.68 (1.17)	3.21 (1.53)	3.82 (0.87)	3.56 (1.50)	3.50 (1.64)	3.00 (1.41)
Cohesion	3.50 (1.19)	3.00 (1.28)	3.53 (1.09)	3.00 (1.32)	3.55 (1.04)	3.36 (1.22)	2.83 (1.72)	4.00 (0.00)
Communication	3.78 (1.24)	3.25 (1.29)	4.00 (1.06)	2.93 (1.27)	4.27 (0.65)	3.48 (1.33)	3.00 (1.67)	4.00 (0.00)
Balanced	1.03 (0.90)	1.08 (0.67)	1.10 (0.84)	0.89 (0.79)	1.09 (0.83)	1.12 (0.83)	0.33 (0.52)	2.00 (0.00)
Satisfied	1.38 (0.83)	1.08 (0.79)	1.23 (0.83)	1.32 (0.72)	1.27 (0.91)	1.20 (0.82)	1.83 (0.41)	1.00 (1.41)

**Table 11:** Mean (standard deviation) of collaboration quality metrics by nationality and education level, and also for teams that include the same or different nationalities.

**Collab. Metrics**	**Nationality**				**Education Level**			
	**USA (16)**	**India (28)**	**Same (19)**	**Differs (15)**	**High Sch. (1)**	**Some Coll (3)**	**College (34)**	**Postgrad. (6)**
Performance	3.19 (1.56)	3.82 (1.19)	3.76 (1.25)	3.13 (1.38)	3.00 (0.00)	4.00 (1.00)	3.62 (1.33)	3.33 (1.86)
Cohesion	3.31 (1.40)	3.39 (1.13)	3.53 (1.17)	3.03 (1.22)	3.00 (0.00)	3.33 (0.58)	3.44 (1.16)	3.00 (1.90)
Communication	3.31 (1.49)	3.82 (1.09)	3.68 (1.11)	3.40 (1.44)	2.00 (0.00)	4.67 (0.58)	3.71 (1.12)	3.00 (1.90)
Balanced	1.06 (0.93)	1.04 (0.79)	1.16 (0.78)	0.83 (0.84)	0.00 (0.00)	1.67 (0.58)	1.15 (0.78)	0.33 (0.82)
Satisfied	1.25 (0.86)	1.32 (0.82)	1.40 (0.76)	1.10 (0.81)	1.00 (0.00)	2.00 (0.00)	1.24 (0.82)	1.33 (1.03)

### 5.7. Impact of Communication Patterns on Perceived Collaboration Quality

We carried out a Spearman correlation test between the communication patterns (the number of occurrences of each communication category for the individual and their team) and the perceived collaboration quality (by individuals^27^).

*Satisfaction* was positively correlated with the *Factual* category (*r* = 0.308, *p* = 0.042, for both the individual and team), also for Defusers (*r* = 0.457, *p* = 0.037, for the individual), but not Lead Experts. So, members seemed more pleased when their team shared more facts, and Defusers particularly when they shared more facts. Satisfaction was also positively correlated with *Planning* but only for Defusers (*r* = 0.437, *p* = 0.047, for the team). It suggests that Defusers were more pleased when the team planned toward the common goal (i.e., defusing the bomb on time).

*Performance* was positively correlated with the *Factual* category only for Defusers (*r* = 0.504, *p* = 0.020, for the team). The more cues were shared among the team members the better Defusers seemed to perceive the team performance.

*Balance* was negatively correlated with the *Uncertainty* category (*r* = –0.378, *p* = 0.011, for the individual), also for Lead Experts (*r* = –0.440, *p* = 0.036; *r* = –0.524, *p* = 0.010, for the individual and team respectively), but not for Defusers. The more questions the Lead Expert asked, and the more questions were asked in the team, the less balanced the Lead Experts seemed to perceive the collaboration.

Finally, *Communication* was positively correlated with the individual *Response* category for Defusers (*r* = 0.457, *p* = 0.028), so the more responsive the Defuser was (e.g., in acknowledging actions they were going to perform), the better they regarded the team communication.

To summarize, several communication categories correlate with perceived collaboration quality, with the role in the team impacting which categories matter. For a good perceived collaboration quality, it seemed important for Defusers to provide facts and neither the team nor the Lead Expert to ask too many questions.

### 5.8. *Post-hoc* Analysis on Impact of Culture

Given our participants mainly came from the USA and India, one may wonder whether there is an impact of culture. Firstly, whilst there is research to show that personality scales can be generalized across cultures (Rolland, 2002; Rammstedt and John, 2007), the distribution in cultures of personality traits differs. Sometimes therefore statine scores (Thorndike, 1982) are used for personality tests to normalize scores based on participants' country of origin. We did not do this, but did consider how the USA and India differ on personality scores, and whether this difference is visible in our participant sample. Table 12 shows the personality scores for the USA and India from the literature, and the scores in our sample. In the literature, the main differences between these countries are on Extraversion and Agreeableness. In our sample, there were significant differences in Openness, Extraversion and Agreeableness between the sample from India and the USA^28^. If we had used stanine scoring normalizing based on the country averages from the literature, the difference between the scores in our sample would have been even bigger (given the averages for India where lower than those for the USA in the literature on these traits, and they already are higher than those for the USA in our sample). We conclude that crowd workers recruited through Mechanical Turk do not represent the average person from their countries. This is not surprising, as for example Burnham et al. (2018) found that Mechanical Turkers from the USA are lower in Extraversion than the general USA population (as was also the case in our sample). To be successful on Mechanical Turk, a certain level of conscientiousness is required (as many tasks require a certain success rate on previous tasks). Similarly, one could imagine that coming from India and working on an American platform requires a certain level of Openness to Experience.

**Table 12:** Mean and standard deviation of the Big Five personality traits in the literature (Bartram, 2013) and in our sample data.

**Data**		**Openness**	**Conscientiousness**	**Extraversion**	**Agreeableness**	**Emotional stability**
Literature	USA	5.29 (2.05)	5.72 (2.03)	5.84 (2.09)	5.34 (1.97)	5.70 (2.05)
Our sample	USA	6.69 (2.19)	8.34 (1.95)	4.13 (2.02)	5.85 (1.83)	5.88 (2.92)
	India	8.55 (1.56)	7.80 (1.89)	6.71 (1.89)	7.43 (1.74)	6.35 (2.02)

There may also be an impact of whether people worked with somebody from their own culture in the task or another culture. We therefore considered whether there was a difference between same nationality teams and teams which differed in nationality on winning the task and on perceptions of collaboration quality (see descriptives in Tables 7, 11, respectively). There was clearly no difference on winning or losing. The perception of collaboration quality seemed slightly better for same nationality teams (with higher means on all measures), but this difference was not statistically significant^29^.

## 6. Discussion, Limitations, and Future Work

### 6.1. Discussion

In this paper, we explored the impact of personality traits, demographics and communication patterns on a virtual collaborative task under time constraints for crowdsourced dyads. Our study observes how the crowd enacts pair-wise roles under pressure, adjusts its communication via chat, and shares common objectives while executing an artificial, video-game-inspired, cooperative time-bound task. Our goal is to use the knowledge from the observations gathered from the study as the basis for future work on AI-supported crowdsourcing of remote emergency response teams. The main results from our exploration, that will need to be verified in follow-on studies, are as follows:

- **Personality and team performance**: minimum Openness to experience seemed to affect the teams' ability to perform under time pressure. Comparatively, teams with higher minimum Openness levels performed better at the remote cooperative task.
- **Communication and team performance**: Communication patterns seemed to matter for team performance: better-performing crowd teams had more Action/Response statements than non-winning teams.
- **Demographics and team performance**: Gender seemed to influence performance, with men slightly more likely to win, however, gender influenced team performance less than the personality trait Openness to experience (minimum).
- **Personality and perception**: Crowd workers' Agreeableness and Conscientiousness likely shaped their perception of the collaboration. Furthermore, dyads that combined people differing in Conscientiousness were perceived by the participants themselves to perform better.
- **Communication and perception**: Communication patterns also seemed to matter for perceived collaboration quality, with the role in the team impacting which categories mattered.

We weigh up these results and connect them with the broader teamwork literature in the coming sections.

#### 6.1.1. Minimum Openness May Impact Teamwork in High-Stress Remote Tasks

Our study demonstrates that the trait of Openness to experience (specifically, its minimum level in a dyadic crowd team) may be a crucial feature for collaboration under pressure and time constraints. This result is novel to the field of team formation since several other studies (Thoms et al., 1996; Barrick et al., 1998; Cogliser et al., 2012; Curşeu et al., 2019) have found that other traits (Conscientiousness first, then Extraversion and Agreeableness) are the most relevant factors affecting team performance. There have been other studies on the effects of personality traits on team performance, such as by O'Neill and Allen (2011) indicating that the trait of Openness is negatively linked with performance *when the team is stable and long-term*, and when it has to perform large analytical tasks such as software engineering. In view of O'Neill and Allen's (2011) study, we read our results as being strongly conditioned by the chosen task type. By highlighting the importance of the trait of Openness, our study helps shed light on the differences that distinguish online *ad-hoc* teams for high-pressure, high-stake tasks, from classical team settings.

Adaptation, as a collateral personality feature of individuals with high Openness to experience, is indeed considered useful in teamwork (Gallivan, 2004), especially in situations of high stress, high-stake and limited time. Moreover, intellectual curiosity with regards to new circumstances is a characteristic observed in people with high Openness to experience (McCrae, 1987); this same trait is closely related to team creativity (Schilpzand et al., 2010). Substantiated by literature (McCrae, 1987; Schilpzand et al., 2010), our results suggest that Openness may act as a more influential factor than task familiarity in determining the success of the team.

#### 6.1.2. Focused Communication Patterns Get the Teams Going

From the results of the analysis of the collaboration, patterns emerge that people who completed the challenge had substantially more Action/Response statements in their chat logs. Thus, they were more effective at communicating with their teammate and promptly came up with clear instructions that helped solve the task on time. Successful participants under pressure used the chat to find a solution right away. Furthermore, winning Defuser predominantly used factual statements. Winning Defusers paid attention to the directives given by their paired teammates (Lead Experts) and responded over the chat by describing where they were at that point in the maze. These results seem to indicate the importance of *focused communication* (with the focus being on efficiency and action clarity), especially when the stakes are high and time-bound. The identification of collaboration patterns has also uncovered tangible clues on how winning individuals intervene during the novel, high-pressure circumstances. Even though communication styles were not communicated explicitly at the start of the task, some participants were more apt at adopting suitable conversational styles as they cooperated and learned from the activity. These findings corroborate other (quasi) longitudinal observations of the long-term impact of risk communication and emergency response measures (Heath and Palenchar, 2000) indicating that citizens are willing to become knowledgeable of emergency response measures and proactively contribute to community relations.

#### 6.1.3. Agreeableness and Conscientiousness Likely Shape the Perception of Collaboration

In our study, highly agreeable people seem to deal better with losing, reflecting more positively on perceived performance, cohesion, and communication. Agreeableness has a social orientation (Bradley et al., 2013) and the trait faceted with trust, altruism, and humility (Matsumoto and Juang, 2016). As highly agreeable people tend to be more sympathetic toward others (Thompson, 2008) and more humble, this may have made them more forgiving toward their teammates and themselves on these aspects. We also found that individuals in teams heterogeneous on Conscientiousness felt better toward the collaboration. Hence, Conscientiousness, at least for high-pressure tasks, is better distributed across teams to improve the perception of teamwork. Making such teams that are heterogeneous in Conscientiousness does not have to be detrimental to actual performance, as shown by our other results as well as Mohammed and Angell (2003). Our result conflicts with that of Gevers and Peeters (2009) who showed that diverse levels of Conscientiousness were negatively linked with teammates' satisfaction. It may be due to the nature of the task since homogeneous high Conscientiousness might have led both the Defuser and the Lead Expert to be overly cautious; however, further studies should investigate the extent of our findings.

#### 6.1.4. Communication Patterns Aligned With Team Roles Matter for the Perception of Collaboration

Communication patterns seemed to matter for the perceived collaboration quality, but this depended heavily on team role. Defusers seemed more satisfied with the collaboration when both themselves and the team used more Factual statements, Lead Experts seemed less satisfied when using Uncertainty statements. These results indicate the importance of team roles and how they are enacted and perceived by teammates. In this instance, the two team roles had distinct and interdependent duties. These reflected the communication patterns that the participants used and preferred (or disliked) above all. In the presence of such distinct team roles, the participants seem to have expected certain communication patterns from their teammates, and these greatly depended on what part of the information they had access to. Defining clear roles is important, as team role clarity improves collaboration (Aritzeta et al., 2005) and communication styles aligned with team roles matter for effective and satisfactory teamwork [as shown in this paper, and in line with (De Vries et al., 2006)]. It may be even more vital in high-pressure tasks with high interdependence.

#### 6.1.5. Gender May Impact Collaboration Though Less Than Personality

Gender seemed to impact team performance, with men slightly more likely to win than women. We considered whether there may have been personality differences. We did not find a statistically significant difference in overall personality traits between genders in this sample. There is some evidence in the literature that there may be a difference in sub-facets of Openness (Weisberg et al., 2011). We also considered whether this is a side effect of the different proportions of men in the sample. More men would result in more teams with men being homogeneous in gender. However, we did not find a significant difference in performance between homogeneous and heterogeneous genders (see Table 7 for descriptives for same gender teams and teams with different genders). Apesteguia et al. (2012) considered the impact of gender on teamwork in an investment game setting. They argued that a decreased performance in homogeneous female teams is explained by differences in decision making, with women being less aggressive and more focused on social sustainability.

We also considered whether gender homogeneity impacted perceptions of collaboration quality (see Table 10 for descriptives). There was a significant impact only on Communication (*post-hoc*, Mann Whitney *U* = 268, *p* < 0.005, Bonferroni corrected), with Communication being appreciated more in same gender teams. As there is a big difference between India and the USA in gender equality (USA is 30^*th*^ (out of 156) in the Global Gender Gap Index (Sharma et al., 2021) compared to India only being 140^*th*^), we also considered the impact of gender homogeneity when teams were diverse in nationality. For teams diverse in gender, there was a significant impact of nationality homogeneity on Cohesion and Balance (*post-hoc*, Mann Whitney *U* = 28, *p* < 0.05, Bonferroni corrected) and similar trends for Communication and Performance (*p* = 0.1 after Bonferroni correction), with all being perceived better for same nationality teams. We considered whether the impact of gender on winning we found may be partially due to women being more likely to have been in diverse gender teams, and collaboration issues having occurred in such teams when the teams were mixed in nationality. However, this was not supported by the data. Further studies are needed to investigate possible cultural factors and their interaction with gender homogeneity. However, given the impact gender may have, gender diversity in teams should be encouraged (Díaz-García et al., 2013).

### 6.2. Limitations

#### 6.2.1. Exploratory Study

As explained above, the study performed was exploratory in nature. Follow-on studies are needed to confirm the results found. The findings from our study can provide the hypotheses for such studies.

#### 6.2.2. Matchmaking System

One of the primary limitations of this study comes from the matchmaking part of the system. We paired participants following a simple first-in-first-out queuing fashion and did not consider user features. This study design choice matched the micro-tasking nature of crowdsourcing and its asynchronous environment, characteristics typical to platforms like Amazon Mechanical Turk. Random matching proved to be an effective solution to the problem of pairing virtual users into *ad-hoc* teams fast and based on availability, and for this reason easily applicable in emergencies. However, this matching limited the control over team formation, rendering the present study observational. For future studies, we plan to test other types of matchmaking criteria. For example, using heuristic algorithms similar to Irvin's Stable Roommate Problem (Irving, 1985) that would assist the matchmaking process according to pre-defined criteria. Other matching systems, such as AI (machine learning and features extraction), could also be used as baselines.

#### 6.2.3. Metrics and Sample

Another limitation of this study is the one associated with the dataset generated from the user outputs and their willingness to give away credible information on their personality traits, demographic data, and experience in the game. We plan to strengthen this area of the research by implementing additional types of secondary data collection systems, such as behavioral, contextual, and sensor data, to help validate and enrich the information gathered about the participants. Different user groups (e.g., students, remote developers, and incident response volunteers) should partake in future studies.

Additionally, our study design did not implement exclusion criteria such as required English proficiency levels nor relied upon pre-screening to filter crowd workers on the basis of their reputation and/or a number of successful HITs. Varying levels of English may have impacted the results. However, most participants reported having completed a College education and the education language at College in all participants' countries (USA, India, UK, Ireland) is English, so we have some confidence that the English level was sufficient not to inhibit communication. We also did not notice clear communication issues due to language in the chats. Nevertheless, future studies will include a test to ensure an appropriate English proficiency level. The absence of pre-screening on English also has a positive aspect, as means our study can be generalized to emergency crises where English is not necessarily the native language whilst still being used for basic virtual communication via chat.

Finally, our sample consisted of predominately male, American, and Indian AMT workers. The sample used for the results likely impacted participants' collaboration and performance. Although we accounted for some of these socio-demographic characteristics (of which gender was significant), we acknowledge the limitations of the dataset derived from the AMT sample. Other types of remote crowd workers from other platforms should experiment with the tool to test for the generalisability of the findings to other portions of the population.

#### 6.2.4. Task, Timer, and Features

The results gathered from the experiments on a single task provide a limited range of conclusions and levels of abstraction to other domains unless other high-stress scenarios could be tested and compared. We plan to implement several types of high-stress tasks. For instance, real-time translation or visual puzzle games would generate more diverse data. They would also quantify the extent to which the choice of task design impacts team collaboration. Another limitation is the lack of manipulation checks for the perceived realism and urgency of the task. It is possible that those workers who did not approach the task seriously might have behaved differently in situations of authentic danger and gravity. Future work should apply similar methodologies and observations to real-life remote emergency situations to be able to test the generalizability of our findings^30^. As part of the development stage, we ran several pilot studies to improve the initial task design and make the instructions clear and understandable for the participating crowd workers.

In the process, we omitted multiple elements present in the original version of the module. We tested different countdowns during the pre-study phase with real users. We settled for a timelimit of 400s as it allowed participants to familiarize themselves with the task interface, chat with one another, and execute the task. Time limits can still be the subject of further testing to evaluate the user's reaction times. We deliberately excluded some of the original elements of the maze module from the video game (i.e., the count of strikes or penalty points for hitting the invisible blocks when crossing the walls, the view of the multiple mazes from the Lead Expert manual, etc.). Tweaking in-game parameters will help uncovering differences in behavior and collaboration that we could not identify by running a single study design. In our experiments, the maze's walls were made invisible to the Defuser while still detectable through object collision. In future studies, and as part of the task improvements, we aim to bring back some of the original features and to assess their significance.

### 6.3. Implications and Future Work

#### 6.3.1. AI Support for Team Formation in Emergency Response

There has been growing research on AI supported team formation, where AI programs allocate workers or learners to teams (Lykourentzou et al., 2013; Odo et al., 2019a). Clearly, the task impacts what team attributes matter for good actual and perceived performance and collaboration. For the emergency task studied in this paper, our primary finding concerns the importance of the trait of Openness to Experience (minimum). When developing an AI group formation system, this can be incorporated (e.g., in the criteria used for automated team formation), ensuring emergency response team have high minimum Openness to Experience, and diverting crowd workers with low Openness to more suitable tasks. Pre-screening and selection procedures are not new to disaster management, but our findings indicate that certain personality traits affect emergency teamwork, and this goes beyond the more common filtering criteria used such as reputation and trust (Javaid et al., 2013). More so, previous research on the effects of personality traits in teamwork did not consider the impact of the task type under stress (Thoms et al., 1996; Barrick et al., 1998; Cogliser et al., 2012; Curşeu et al., 2019), particularly in cases of emergency response. The sample of crowd workers used in this study helped us understand how pairs of non-familiar and dispersed users act together when presented with an unseen challenge. By utilizing AI to infer the crowd's attributes through their interactions, intelligent systems can learn to adjust to their needs and capabilities in times of emergency and suggest collaborators for a better fit.

The results from this specific approach are beneficial to the crowdsourcing and online work fields that are becoming ever so relevant due to recent and significant changes in the way we live and work. In the Ukrainian conflict of 2022, volunteers of remote rescue operations based in the USA allocated buses to civilians making requests for help online and helping save countless lives (Mark et al., 2022). By remote communication and real-life GPS updates, citizens from far away aided the evacuation of many citizens by identifying grounds hit by shelling and bombing. Following tragic examples like this one, researchers and industry can weigh the power of AI to aid the team formation process of remote emergency crowd teams and assist with organizing rescue units during high-stress, life-threatening situations.

#### 6.3.2. Conversational AI Support for Remote Emergency Response Teams

The analysis of the communication patterns clearly indicated that not all teams focused on the task execution correctly since some adopted less-than-optimal communication strategies. Our results provide insights into which communication acts may be important which can be used by an AI system to monitor and moderate remote collaboration and intervene when needed. With the implementation of machine learning models, future crowdsourcing tools specialized in emergency response can augment the chat functionality by deploying conversational AI (Battineni et al., 2020) (as an example) moderating users' communication patterns. With the stark improvements in Natural Language Generation, Understanding, and Processing, and the increasingly reduced costs of production thanks to open-source software community (Adamopoulou and Moussiades, 2020), most forms of crowdsourced self-organized teams (e.g., neighborhood watch Bakker et al., 2012) could themselves incorporate, maintain, and improve machine learning models for emergency response conversational AI initially trained on annotations and knowledge such as the one we present.

We note that personality traits seemed to affect the perception of the collaboration. Although system evaluations usually pursue metrics similar to ours (e.g., effectiveness, efficiency, and reliability), team performance is only part of the equation. While a team can successfully reach a goal on time, the perception of teamwork is not always directly proportional to that outcome. What individuals think, interpret, and how they respond to changes can be conditioned by personality factors. In this study, we observe the interaction between personality and communication patterns. With defined team roles and interdependency, people with certain personality traits are likely to expect from others certain communication styles. Further, personality seems to have determined the propensity for more or less rigor and clarity in the communication. Considering the numerous variables at play and the increased reliance on crowdsourcing for rescue operations and emergency response (Marc Cieslak, 2022), we advocate for the development of adaptive and personalized intelligent systems. AI-aided emergency response can provide support and knowledge to teams according to the individual and group needs to alleviate stress and improve community participation. Emotional support could be tailored to the individuals and made accessible and private in critical emergency settings addressing the lack of sensemaking and trust emerging from periods of stress, trauma, and danger.

## 7. Conclusion

In this study, 60 crowd dyads collaborated in a high-pressure, computer-mediated task. The study required them to play complementary roles in a time-bounded critical scenario. We explored the possible impact of the participants' personality, socio-demographic factors, and communication patterns on team performance and perceived collaboration quality. Results from our exploratory study suggest that teams scoring high on the personality trait of Openness (meaning that the minimum Openness of winning teams was higher than in the losing teams) performed overall better in the execution of this high-pressure task. The analysis of the team communication patterns suggest that teams communicating more through action-response loops were more likely to win the game. Different levels of Agreeableness and Conscientiousness likely shaped the perception of collaboration with highly agreeable people coping better with losing. Teams heterogeneous on Conscientiousness seemed to feel better about the teamwork. Communication patterns seemed to matter for the perceived collaboration quality, but this was highly role-dependent, showing that communication styles aligned with team roles matter for effective and satisfactory teamwork. We can learn from these exploratory results that the perception of the collaboration may differ depending on personality traits and the communication patterns shared among remote teammates. So, intelligent crowdsourcing-aided emergency response technology may need to consider individuals' viewpoints and provide adequate support for the crowd needs. Our findings support future research on computer-based collaboration under pressure. It shows ways to tailor the development of AI as accessible support in crowdsourcing emergency response aiding with team formation, conversational support, and adaptation. Future work will confirm the findings and evaluate other types of high-stress tasks, time limits, and parameters for team formation to advance the findings presented here.

## Data Availability Statement

The raw data supporting the conclusions of this article will be made available by the authors, without undue reservation.

## Ethics Statement

Ethical review and approval was not required for the study on human participants in accordance with the local legislation and institutional requirements. The patients/participants provided their written informed consent to participate in this study.

## Author Contributions

All authors listed have made a substantial, direct, and intellectual contribution to the work and approved it for publication.

## Conflict of Interest

The authors declare that the research was conducted in the absence of any commercial or financial relationships that could be construed as a potential conflict of interest.

## Publisher's Note

All claims expressed in this article are solely those of the authors and do not necessarily represent those of their affiliated organizations, or those of the publisher, the editors and the reviewers. Any product that may be evaluated in this article, or claim that may be made by its manufacturer, is not guaranteed or endorsed by the publisher.

## Footnotes

^1^Emotional Stability is often replaced in literature by its opposite Neuroticism.

^2^studies have been conducted on construct MBTI validity and test-retest reliability (including a meta-study by Capraro and Capraro (2002) which showed good results), others have argued that there are scientific limitations to these studies, the use of MBTI, and its underlying theory (e.g., Boyle, 1995; Pittenger, 2005; Stein and Swan, 2019).

^3^AMT worker's population is composed primarily of Indian and American nationalities, followed by Chinese, British, and Philippino (Difallah et al., 2018). The gender is slightly predominantly female within the American sample and more male in other countries (Difallah et al., 2018). Its population average age is less than the world population average, as most AMT workers were born after the 1990's (Difallah et al., 2018).

^4^Also requiring considerably longer instructions and the introduction of manipulation checks to ensure instructions were read which further adds to task complexity.

^5^as the BFI-10 uses 5-point Likert scales one could argue that the data is ordinal, but given a total is calculated per trait we will regard it as interval.

^6^Free text entry, values provided here are those used by participants.

^7^Reverse scored means that a 1 is changed into 5, 2 into 4, 4 into 2, and 5 into 1.

^8^Test-retest correlations suggest acceptable reliability on a Likert scale of 1 (Disagree strongly) to 5 (Agree strongly). As prior studies have shown, the correlations of this instrument with other Big Five instruments, its correlations with self-and peer-ratings, and its associations with socio-demographic variables suggest good validity of the BFI-10 inventory (Rammstedt and John, 2007).

^9^Reversed means that a score of 1 is changed into 5, 2 into 4, 4 into 2, and 5 into 1.

^10^which in the Group Recommender community are called, respectively, Least Misery and Most Pleasure.

^11^Personality traits likely differ on whether a high (or low) trait level positively or negatively impacts team performance. Using both minimum and maximum ensures this is no longer an issue.

^12^For teams of two, the use of standard deviation is equivalent to the use of numerical difference. We opted for standard deviation to build on the work by Odo et al. (2019b) and for generalizability to larger groups.

^13^Team, rather than individual level was used since it is usually the combination and interaction among individuals' personalities that affects the team outcome, as evidenced by multiple studies [e.g., see Gilley et al.'s (2010) comprehensive review].

^14^Less conservative corrections such as Tukey are not possible due to the data often not meeting normality assumptions.

^15^Additionally, as many measures were not independent, Bonferroni corrections would also have been less appropriate.

^16^Including no impact of Neuroticism or differences of standard deviation.

^17^We only performed the logistic regression with the minimum metric as minimum Openness was the only variable that was significant in the Mann-Whitney test, hence avoiding running multiple tests increasing the chances of Type I error.

^18^Hosmer and Lemeshow test was not significant, thus, the model assumptions were met.

^19^Namely prefer not say for gender, and British and Irish for nationality, all with frequency 1.

^20^This also means that Agreeableness needs to be considered when interpreting indirect measures of team collaboration quality as it may make them a less accurate reflection of actual collaboration.

^21^This seems more likely than that Agreeableness influenced the performance, communication, and cohesion itself, certainly given the lack of correlations for winners.

^22^Spearman correlations average Openness: *r =* 0.398, *p =* 0.02; minimum Openness *r =* 0.410, *p =* 0.02.

^23^Spearman correlation: *r =* 0.400, *p =* 0.02.

^24^Spearman correlations Performance: *r =* 0.644, *p* < 0.0001; Communication quality: *r =* 0.492, *p =* 0.003; Cohesion *r =* 0.403, *p =* 0.02; Balance: *r =* 0.448, *p =* 0.008; Satisfaction: *r =* 0.417, *p =* 0.01.

^25^There was also a significant Spearman correlation for minimum Conscientiousness: *r =* –0.423, *p =* 0.01.

^26^There was a significant difference for education level on balance, but given the small numbers in all groups.

^27^Given the low number of teams were both members responded, we used the perceived collaboration quality at the individual level only.

^28^*Post-hoc* test, Mann-Whitney *U* = 811.5, *U* = 611.0, *U* = 933,5 respectively, with *p* < 0.001 (and still significant if Bonferroni corrected).

^29^Perceived performance was significant at *p* < 0.05, but not when Bonferroni correction was applied.

^30^However, there are clear ethical issues with this.
